# *PKM* splice-switching ASOs induce upregulation of dual-specificity phosphatases and dephosphorylation of ERK1/2 in hepatocellular carcinoma

**DOI:** 10.1016/j.jbc.2025.108345

**Published:** 2025-02-22

**Authors:** Dillon M. Voss, Alexander J. Kral, GeunYoung Sim, Raditya Utama, Kuan-Ting Lin, Chris Cizmeciyan, Balazs Schafer, Patrick J. Cunniff, Christopher R. Vakoc, Marvin H. Caruthers, Lopa Mishra, Adrian R. Krainer

**Affiliations:** 1 Cold Spring Harbor Laboratory, New York, USA; 2 Renaissance School of Medicine, Stony Brook University, Stony Brook, New York, USA; 3 Department of Microbiology and Immunology, Renaissance School of Medicine, Stony Brook University, Stony Brook, New York, USA; 4 Department of Biochemistry, University of Colorado, Boulder, Colorado, USA; 5 The Institute for Bioelectronic Medicine, The Feinstein Institutes for Medical Research, Division of Gastroenterology and Hepatology, Northwell Health, New York, USA

**Keywords:** cancer, antisense RNA, pyruvate kinase, hepatocellular carcinoma, dual-specificity phosphoprotein phosphatase

## Abstract

Pyruvate kinase muscle isoform 2 (PKM2) is preferentially expressed in nearly all cancers. It primarily functions as the last enzyme in glycolysis but has other reported noncanonical functions, including recruiting transcription factors to oncogenes and phosphorylating proteins. We previously described antisense oligonucleotides that disrupt alternative splicing of *PKM* pre-mRNA (PKM-ASOs), resulting in a PKM2-to-PKM1 isoform switch in hepatocellular carcinoma (HCC), which reduces HCC growth *in vitro* and *in vivo*. PKM1 has higher enzymatic activity than PKM2, which potentially drives metabolism away from macromolecule synthesis, and may explain decreased HCC growth upon PKM-ASO treatment. As PKM-ASOs also reduce PKM2 levels, how *PKM* splice-switching inhibits HCC cell proliferation was unclear. Here, we characterized the individual consequences of altering PKM1 or PKM2 protein levels in HCC and observed that reducing PKM2 alone was sufficient to decrease HCC cell proliferation, whereas overexpressing PKM1 had no effect. Moreover, increasing PK activity *via* a small-molecule PKM2 activator had no effect on HCC cell proliferation, suggesting that PKM-ASOs affect PKM2's nonmetabolic functions. Transcriptomic and RT-qPCR analyses of HCC cells treated with PKM-ASO or PKM2-siRNA revealed upregulation of dual-specificity phosphatase 2 (DUSP2) and other related DUSPs, which act directly on ERK1/2 in the MAPK signaling pathway. Luciferase reporter assays demonstrated that PKM-ASO treatment activated the *DUSP2* promoter, which correlated with decreased ERK1/2 phosphorylation. Lenvatinib is a second-line HCC therapy that indirectly reduces ERK1/2 phosphorylation, and combined treatment with PKM-ASOs inhibited proliferation of HCC cells more than either treatment alone. In summary, our results reveal a mechanism by which PKM-ASOs affect PKM2 dependency in HCC.

Pyruvate kinase (PK) catalyzes the last step of glycolysis, converting phosphoenolpyruvate to pyruvate (1). Concomitantly, the phosphate of phosphoenolpyruvate is transferred to a molecule of ADP, resulting in the formation of ATP. Four versions of PK are expressed in humans, each with unique enzyme kinetics. PK-liver and PK-red blood cell are isoforms expressed from the same *PKLR* gene and are present almost exclusively in the liver or in red bloods cells, respectively, reflecting the use of tissue-specific promoters. Pyruvate kinase muscle isoform 1 (PKM1) and pyruvate kinase muscle isoform 2 (PKM2) are mutually exclusive alternatively spliced isoforms expressed from the *PKM* gene and are regulated in both tissue-specific and metabolism-specific contexts (1). Notably, the PKM2 isoform is upregulated in nearly all cancers (1). PKM2's enzymatic activity is much lower than that of its PKM1 counterpart, due to the fact that PKM2 is predominantly an inactive dimer, whereas PKM1 forms a constitutively active tetramer (1). There are numerous metabolites that induce PKM2 tetramerization, notably fructose-1,6-bisphosphate. However, fructose-1,6-bisphosphate release and PKM2 dimerization occur when the K433 residue is bound to tyrosine-phosphorylated proteins, many of which are downstream from various oncogenic signaling pathways (2, 3).

Although PKM2 is upregulated in nearly all cancers, the exact mechanism by which it provides a growth advantage to cancer cells remains unclear. One model proposed that PKM2 aids in shunting key upstream glycolytic metabolites into various macromolecule-synthesis pathways to provide sufficient building blocks to sustain rapid cell division (4). Other reports provided evidence to suggest that dimeric PKM2 plays a direct role in upregulating various oncogenic signaling pathways by translocating to the nucleus and recruiting transcription factors to oncogenes (1, 5, 6, 7). Further complicating our understanding of the role of PKM2 in oncogenesis is the fact that different cancers have varying metabolic demands and may harbor unique gene mutations. Therefore, it is possible that both proposed roles for PKM2 are valid, but their potential contribution to cancer is context-specific. Regardless of the mechanism, numerous reports have shown that reducing PKM2 expression is associated with a decrease in tumor growth in various cancers, which makes PKM2 a potential therapeutic target (4). However, there are currently no approved PKM2-targeting therapies to treat cancer.

Our lab previously developed antisense oligonucleotides (ASOs) that redirect alternative splicing of *PKM* pre-mRNA in cancer (PKM-ASOs), which results in decreased PKM2 and increased PKM1 (8, 9, 10). We further showed that our PKM-ASOs reduce tumor growth in two preclinical murine models of hepatocellular carcinoma (HCC) (10). Moreover, we detected PKM-ASOs within the liver and fibrovascular stroma of the liver tumors, without detectable toxicity in the spleen, lung, and kidney (10). Importantly, off-target effects on adjacent liver tissue were minimal, and on-target toxicity in the liver is not expected, because normal hepatocytes rely on *PKLR* (10). Given these promising results, we sought to further characterize the effects and the underlying mechanisms of PKM-ASOs in the context of HCC.

In this study, we conducted RNA-seq analysis on HCC cells treated with PKM-ASOs. We compared and validated gene-expression changes from HCC cells treated with PKM-ASOs to those in cells treated with either an siRNA-targeting PKM2 or in cells overexpressing exogenous PKM1. This approach allowed us to separate the effects of decreasing PKM2 *versus* increasing PKM1, both of which take place upon ASO-induced PKM splice-switching. Our data suggest that the therapeutic effect of our PKM-ASOs primarily reflects downregulation of PKM2, which in turn induces increased expression of DUSP2 and related dual-specificity protein phosphatases that dephosphorylate ERK1/2 kinases.

## Results

### Downregulation of PKM2, but not increased PKM1, reduces HCC cell line proliferation *in vitro*

**Figure 1:**
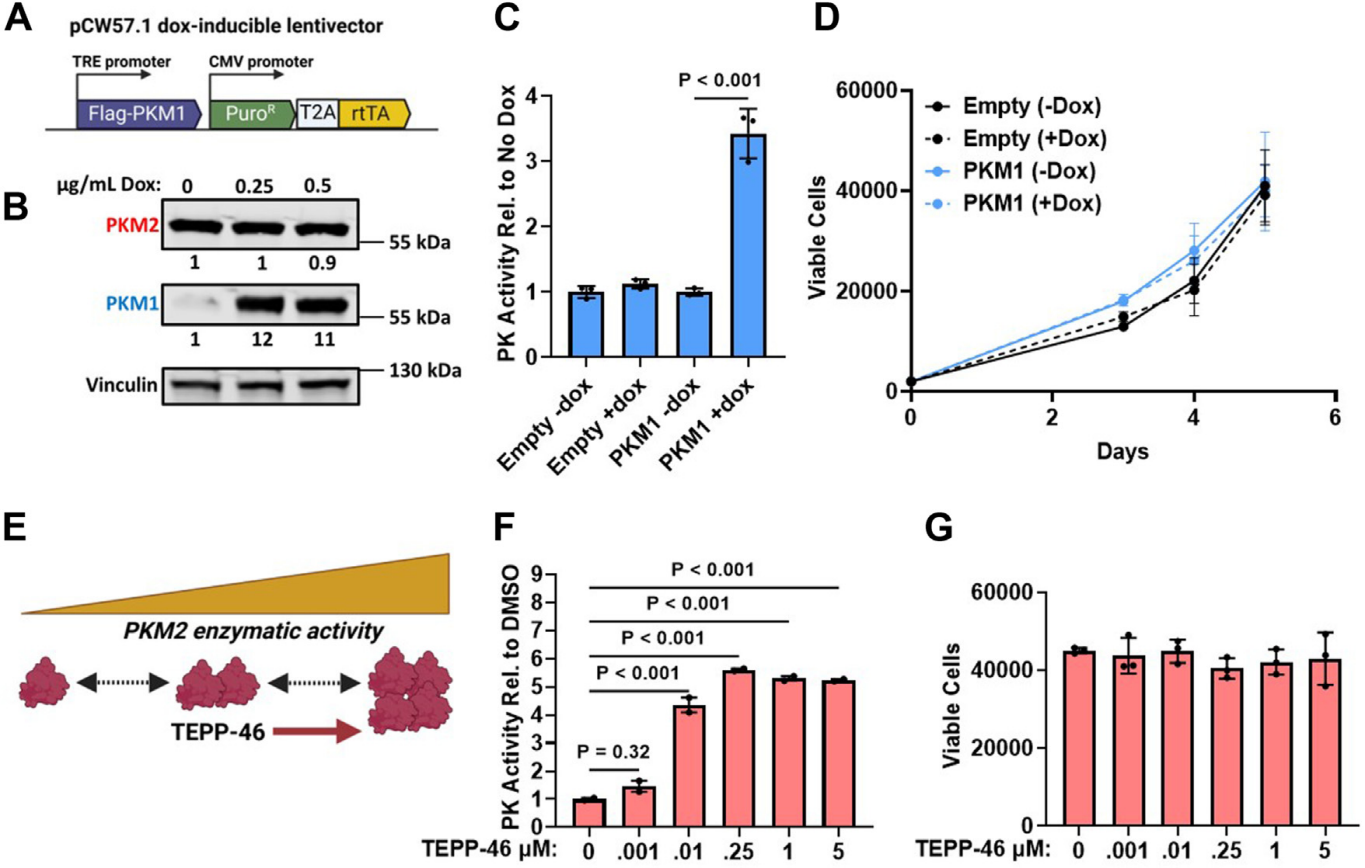
**Increasing PK activity does not alter HCC cell proliferation.***A*, diagram of the pCW57.1-Flag-PKM1 lentiviral vector. *B*, Western blotting analysis of PKM1 protein levels in the SNU449-Flag-PKM1 (SNU449-PKM1OE) cell line after treatment with the indicated concentration of doxycycline for 5 days. Cells were treated with dox on days 0, 2, and 4. Quantification of band intensities is shown below; bands were normalized to vinculin and to the no-dox control. *C*, PK activity in SNU449-PKM1OE cells treated with 0.25 μg/ml dox on days 0, 2, and 4. PK activity was quantified on day 5. *D*, viable cells were counted using ViaCount and flow cytometry. SNU449-PKM1OE or empty-vector cells were treated as in (*C*). *E*, diagram of TEPP-46 inducing tetramerization of PKM2, which leads to increased enzyme activity. *F*, PK activity on day 5 of SNU449 cells treated with TEPP-46 on days 0, 2, and 4, at the indicated concentrations. *G*, viable SNU449 cells were counted on day 5 using ViaCount and flow cytometry. Cells were treated with TEPP-46 as in (*F*). One-way ANOVA was performed with Tukey's multiple comparison *post hoc* test for data in (*C*, *D*, *F*, and *G*). Data in (*C* and *D*) represent the average of three independent biological replicates ± SD. Data in (*F* and *G*) represent the average of two independent biological replicates ± SD. There was no significant change detected between treatment groups in (*D* and *G*).

We previously showed that *PKM* splice-switching ASOs (PKM-ASOs) upregulate PKM1 and downregulate PKM2, which results in the decreased proliferation of HCC cells (10). To determine PKM1-specific effects on HCC proliferation, we generated derivatives of two different HCC cell lines (SNU449 and SNU398) and one hepatoblastoma cell line (HepG2) that stably express a doxycycline-inducible Flag-PKM1 cDNA (PKM1-OE) (Fig. 1*A*). We observed that treating cells with 0.25 μg/ml doxycycline (dox) for 5 days was sufficient to markedly induce PKM1 protein levels in all three cell lines, relative to nontreated cells (Figs. 1*B* and S1*A*). Moreover, dox-treatment for 5 days resulted in a significant increase in PK activity in all cell lines (Figs. 1*C* and S1*B*). However, automated cell counting with ViaCount revealed that dox-inducible expression of PKM1 did not alter cell proliferation in the three cell lines (Figs. 1*D* and S1*C*). Notably, the 12-fold increase in PKM1 observed in the dox-induced SNU449 PKM1-OE cells was ∼3-fold higher than the PKM1 increase in SNU449 cells treated with PKM-ASOs (see below).

To further test the effect of increasing PK activity on HCC proliferation, we utilized the small-molecule PKM2 activator TEPP-46 (11, 12). TEPP-46 binds directly to PKM2, resulting in its tetramerization, which increases PK activity to a level comparable to that of PKM1 (11) (Fig. 1*E*). We treated SNU449, Huh7 (another HCC cell line), and HepG2 cells with various concentrations of TEPP-46 for 5 days and measured both PK activity and cell proliferation. We found that a relatively low concentration of 10 nM TEPP-46 had a maximal effect on PKM2 tetramerization in all three cell lines, considering that increasing the dose up to 5 μM only minimally further increased PK activity (Figs. 1*F* and S2*A*). The average increase in PK activity at the 10 nM dose was 4-fold for SNU449, 18-fold for Huh7, and 32-fold for HepG2 (Figs. 1*F* and S2*A*). Importantly, there were no detectable changes in proliferation as a result of increased PK activity in any of the three cell lines (Figs. 1*G* and S2*B*).

These data show that increasing PK activity either *via* upregulation of exogenous PKM1 or activation of PKM2 does not alter proliferation of the liver-cancer cell lines. Therefore, although PKM-ASOs upregulate PKM1 and increase PK activity, it is unlikely that this effect alone directly impacts HCC cell proliferation.

**Figure 2:**
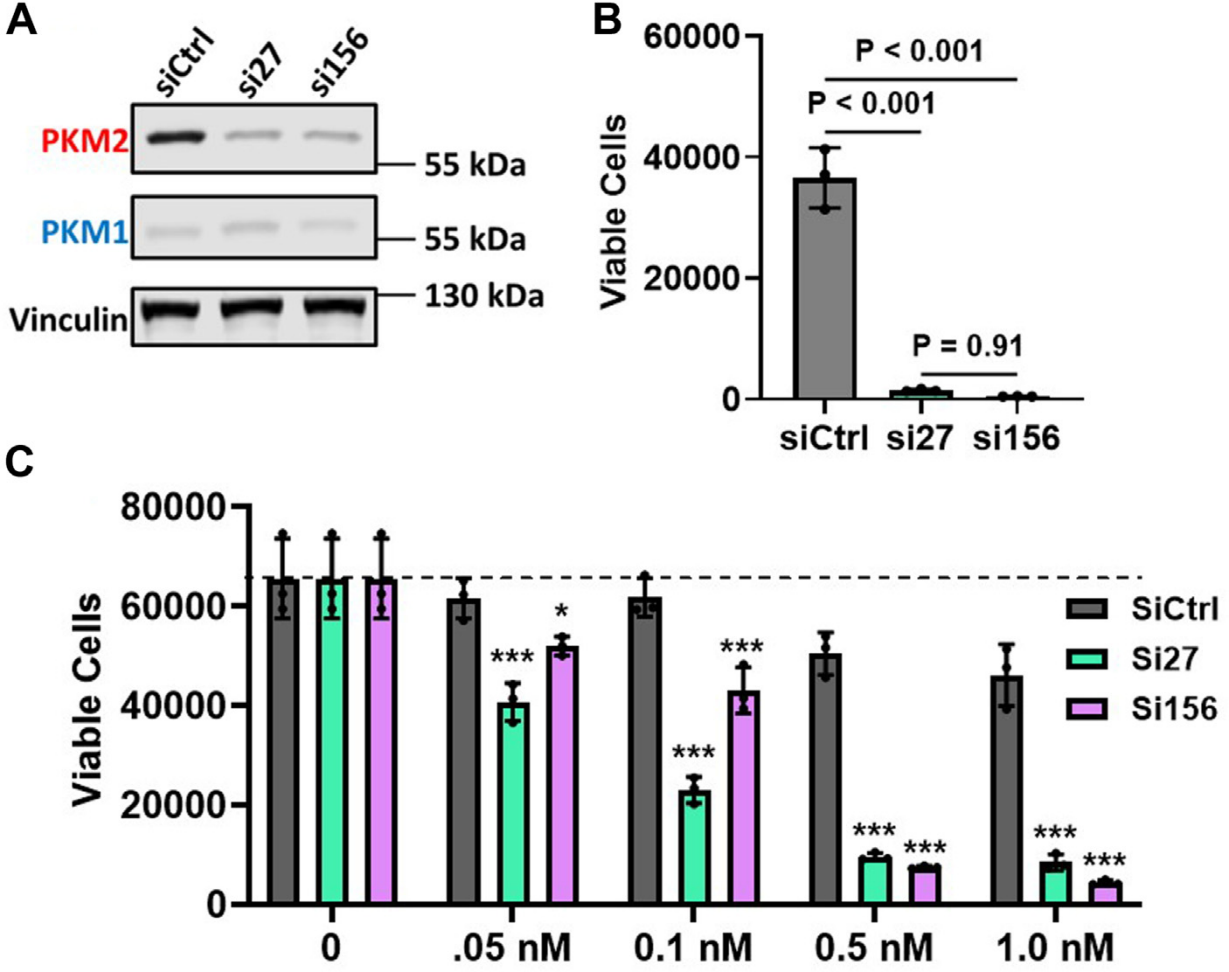
**siRNA knockdown of PKM2 inhibits HCC cell proliferation.***A*, Western blotting analysis of PKM1 and PKM2 in SNU449 cells 2 days after transfecting 5 nM siRNA. *B*, viable cells counted with flow cytometry. Cells were transfected with 5 nM siRNA and incubated for 5 days, with repeat transfection on day 2. *C*, viable cells counted and treated with various concentrations of siRNA as in (*B*). The bar graphs in (*B*) and (*C*) represent the average of three independent biological replicates ± SD. For data in (*B* and *C*), one-way ANOVA was performed with Tukey's multiple comparison *post hoc* test. For (*C*), statistical analysis was performed for each siRNA concentration displayed. ∗*p* < 0.05 and ∗∗∗*p* < 0.001.

To determine the effects of PKM2-specific knockdown on HCC proliferation, we utilized two siRNAs (si27 and si156) that were previously shown to decrease expression of PKM2 *via* binding *PKM* exon 10 (13). We transfected 5 nM of si27, si156, or a nontargeting siRNA (siCtrl) into SNU449, Huh7, and HepG2 cells and assessed cell proliferation using ViaCount and automated cell counting at 5 days post-transfection. Western blotting analysis of PKM1 and PKM2 in cells treated with either of the PKM2 siRNAs showed decreased PKM2 protein levels relative to siCtrl and no change in PKM1 protein levels (Figs. 2*A* and S3*A*). The proliferation assay showed a statistically significant reduction in cell proliferation after treatment with either si27 or si156, relative to siCtrl, in all three liver-cancer cell lines (Figs. 2*B* and S3*B*). Additionally, we quantified proliferation in response to varying doses of siPKM2 that spanned from 50 pM to 1 nM and observed a dose-dependent decrease in proliferation (Figs. 2*C* and S3*C*). We conclude that downregulation of PKM2 alone is sufficient to reduce HCC proliferation *in vitro*. Moreover, these results strongly suggest that PKM-ASOs alter HCC proliferation primarily *via* downregulation of PKM2.

### Transcriptomic analysis of HCC cells treated with either siPKM2 or PKM-ASOs reveals altered expression of DUSPs

Given that both siPKM2 and PKM-ASOs downregulate PKM2 and alter HCC proliferation, we next sought to identify and compare the changes in gene expression resulting from either treatment, by performing RNA-seq analysis. To this end, we utilized PKM-ASOs with thiophosphoramidate morpholino chemistry (TMOs) (Fig. S4*A*) (14). The sequences of TMO1 and TMO2 are identical to the sequences of the 2′, 4′- constrained 2′-O-ethyl (cEt) ASO1 and ASO2 that we previously reported (10), respectively (Fig. S4*B*). We observed that PKM-TMOs and cEt-ASOs elicited similar extents of *PKM* splice-switching when transfected at 60 nM into HepG2 cells for 48 h (Fig. S4, *C*–*E*). We also observed that 60 nM transfection of either TMO1 or TMO2 induced *PKM* splice-switching in both Huh7 and SNU449 cells and significantly reduced cell proliferation within 4 days, relative to a scrambled-sequence TMO (TMO-Ctrl) (Fig. S4, *F*–*I*). The decrease in proliferation in SNU449 cells treated with TMO1 or TMO2 was associated with a relative 4-fold and 5-fold increase in PKM1, respectively, whereas dox-induction in SNU449-PKM1OE cells (Fig. 1*B*) resulted in a 12-fold increase in PKM1 but did not alter cell proliferation. Moreover, treatment with either TMO1 or TMO2 reduced PKM2 levels in SNU449 cells by 80% and 60%, respectively (Fig. S4*G*), whereas there was no change in PKM2 levels in dox-induced SNU449-PKM1OE cells (Fig. 1*B*). Taken together, these results further suggest that the decrease in cell proliferation from PKM-TMO treatment is primarily due to downregulation of PKM2.

We performed RNA-seq analysis on our dox-inducible SNU449-PKM1OE cell line after 48 h treatment with 5 nM siPKM2 or 60 nM TMO2 or 0.25 μg/ml dox (Fig. S5*A*). We then identified differentially expressed genes (DEGs) between the various treatment groups and their respective controls. We considered DEGs with a false discovery rate–adjusted *p*-value of less than 0.05 and a Log2 fold-change greater than 0.585 (*i.e.*, 1.5-fold). Remarkably, except for *PKM*, we were unable to detect any DEGs between dox-treated and untreated cells. In contrast, in the comparisons of siPKM2 *versus* siCtrl and TMO2 *versus* lipofectamine control (lipo-Ctrl), we observed 1521 and 845 unique DEGs, respectively (Fig. S5*B*). Importantly, we identified 264 DEGs shared between the siPKM2-treated and TMO2-treated groups (Fig. S5*B* and Table S1).

We then utilized the Database for Annotation, Visualization, and Integrated Discovery (DAVID) to perform functional annotation clustering on the list of 264 DEGs shared between the siPKM2-treated and TMO2-treated groups. This analysis yielded a single cluster with an overall enrichment score of 2.45, which comprises various MAPK-related terms. Correction for multiple-hypothesis testing narrowed down our list to three terms that all referred to MAPK tyrosine phosphatase activity (Fig. S5*C*). These three terms comprise an identical list of four genes, namely the dual-specificity phosphatase genes *DUSP2*, *DUSP4*, *DUSP5*, and *DUSP6*. Notably, all four of these DUSPs act directly on ERK1/2 of the MAPK signaling pathway, promoting dephosphorylation of ERK1/2 and suppression of the pro-proliferative component of MAPK signaling (15). In our RNA-seq analysis dataset, we observed that all four DUSPs were upregulated in both our siPKM2-treated and TMO2-treated cells, relative to their respective controls (Fig. S5*D*).

**Figure 3:**
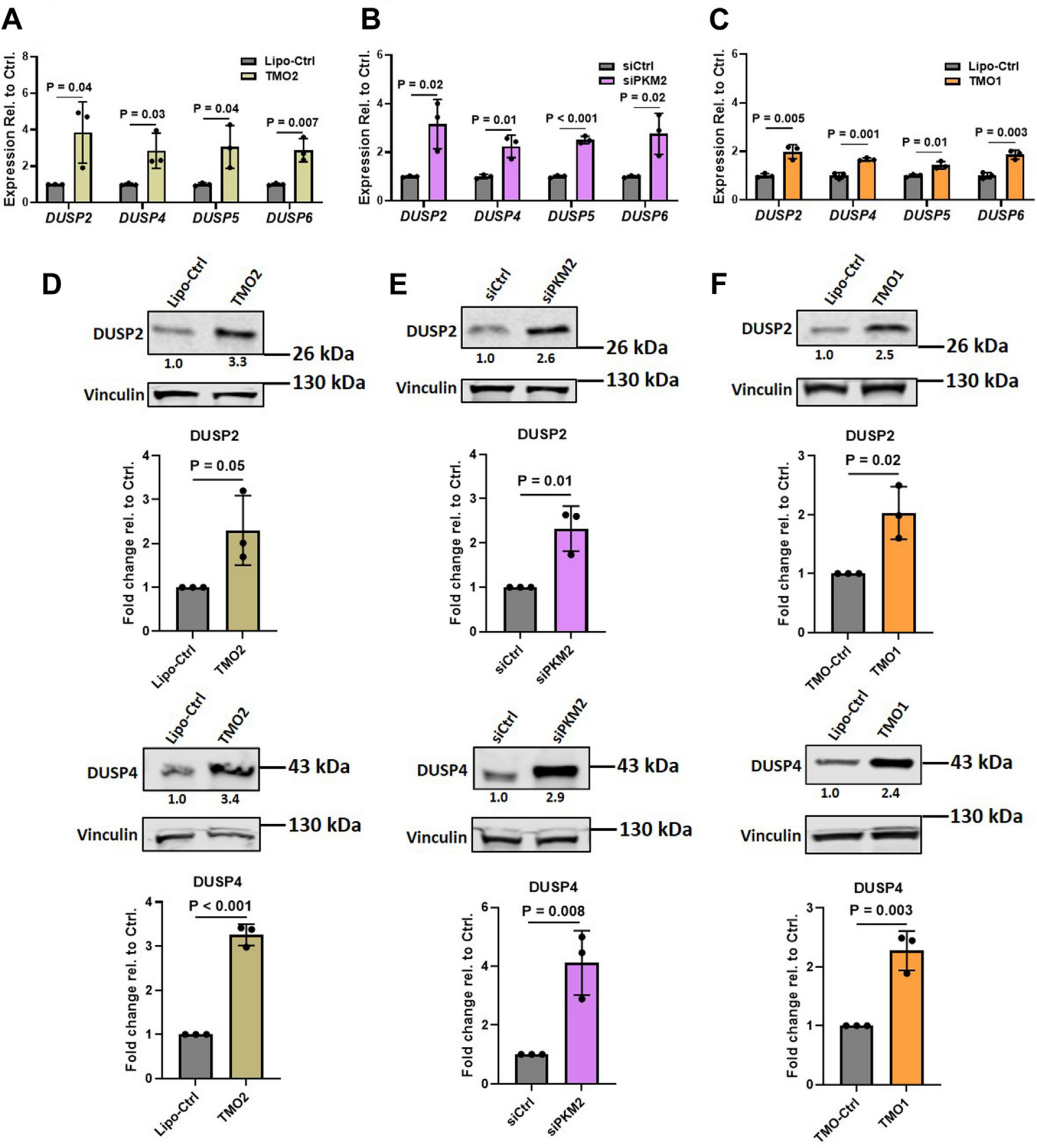
**SNU449 cells treated with siPKM2 or PKM-TMO show increased expression of multiple DUSPs.***A*, RT-qPCR quantitation shows the extent of *DUSP* mRNA increase following transfection of SNU449-PKM1OE cells with 60 nM TMO2 for 48 h. All tested transcripts were normalized to the *ACTB* mRNA level. Relative expression to cells treated with lipofectamine-only (Lipo-Ctrl) is plotted. *B*, RT-qPCR quantitation shows the extent of increased *DUSP* mRNA following transfection of SNU449-PKM1OE cells with 5 nM si157 or siCtrl for 48 h, with normalization as in (*A*); relative expression to cells treated with siCtrl is plotted. *C*, RT-qPCR quantitation shows the extent of increased *DUSP* mRNA following transfection of SNU449-PKM1OE cells with 60 nM TMO1 for 48 h, with normalization as in (*A*); relative expression to cells treated with Lipo-Ctrl is plotted. *D*, Western blotting analysis of DUSP2 and DUSP4, following transfection for 48 h with either TMO2 or Lipo-Ctrl, with quantification of band intensities shown below; bands were normalized to vinculin and to the Lipo-Ctrl. *E*, Western blotting analysis of DUSP2 and DUSP4 from cells treated as in (*B*); bands were normalized to vinculin and to the siCtrl. *F*, Western blotting analysis of DUSP2 and DUSP4 from cells treated as in (*C*); bands were normalized to vinculin and to the Lipo-Ctrl. The bar graphs in (*A*–*F*) represent the average of three independent biological replicates ± SD. A two-sided *t* test was performed individually for each gene shown.

We next carried out downstream target validation of the RNA-seq analysis by performing reverse transcription-quantitative polymerase chain reaction (RT-qPCR) and Western blotting analyses of the various DUSPs of SNU449 cells transfected for 48 h with 60 nM TMO1 or TMO2 or 5 nM siPKM2. The results revealed statistically significant increases of ∼3-fold in *DUSP2*, *DUSP4*, *DUSP5*, and *DUSP6* mRNA levels for TMO2-treated and siPKM2-treated cells and ∼2-fold for TMO1-treated cells (Fig. 3, *A*–*C*). Western blotting analysis of DUSP2 and DUSP4 revealed a >2-fold increase in both proteins in all three treatment groups (Fig. 3, *D*–*F*). We were unable to detect an increase in DUSP5 or DUSP6 protein levels under these conditions, so we conclude that treatment with either siPKM2 or PKM-TMOs primarily upregulates DUSP2 and DUSP4. Additionally, we found that treatment of SNU398 and Huh7 cells with TMO1 upregulated DUSP2 mRNA, but not DUSP4, 5, or 6 mRNAs (Fig. S6*A*).

Given the inverse correlation we observed between PKM2 expression and expression of various DUSPs *in vitro*, we next sought to determine whether this correlation also occurs in human HCC tumors. To this end, we quantified gene expression of *PKM*, *DUSP2*, *DUSP4*, *DUSP5*, and *DUSP6*, using the liver hepatocellular carcinoma cohort of The Cancer Genome Atlas dataset. PKM1 and PKM2 isoforms only differ in the sequences corresponding to mutually exclusive exons 9 and 10, respectively, so most RNA-seq reads do not distinguish between these isoforms. However, as PKM2 is the predominant isoform in HCC, total *PKM* expression serves as a proxy for PKM2 levels. Surprisingly, we observed a positive correlation between *PKM* expression in tumors and expression of *DUSP2*, *DUSP4*, and *DUSP5* (Fig. S6, *B*–*E*). These results suggest that whereas acute decreases in PKM2 result in increased DUSP levels *in vitro*, other mechanisms likely influence DUSP expression throughout HCC tumor development and maintenance (See Discussion).

### Downregulation of PKM2 *via* siPKM2 or PKM-TMO in HCC cells induces DUSP2 expression *via* promoter activation

**Figure 4:**
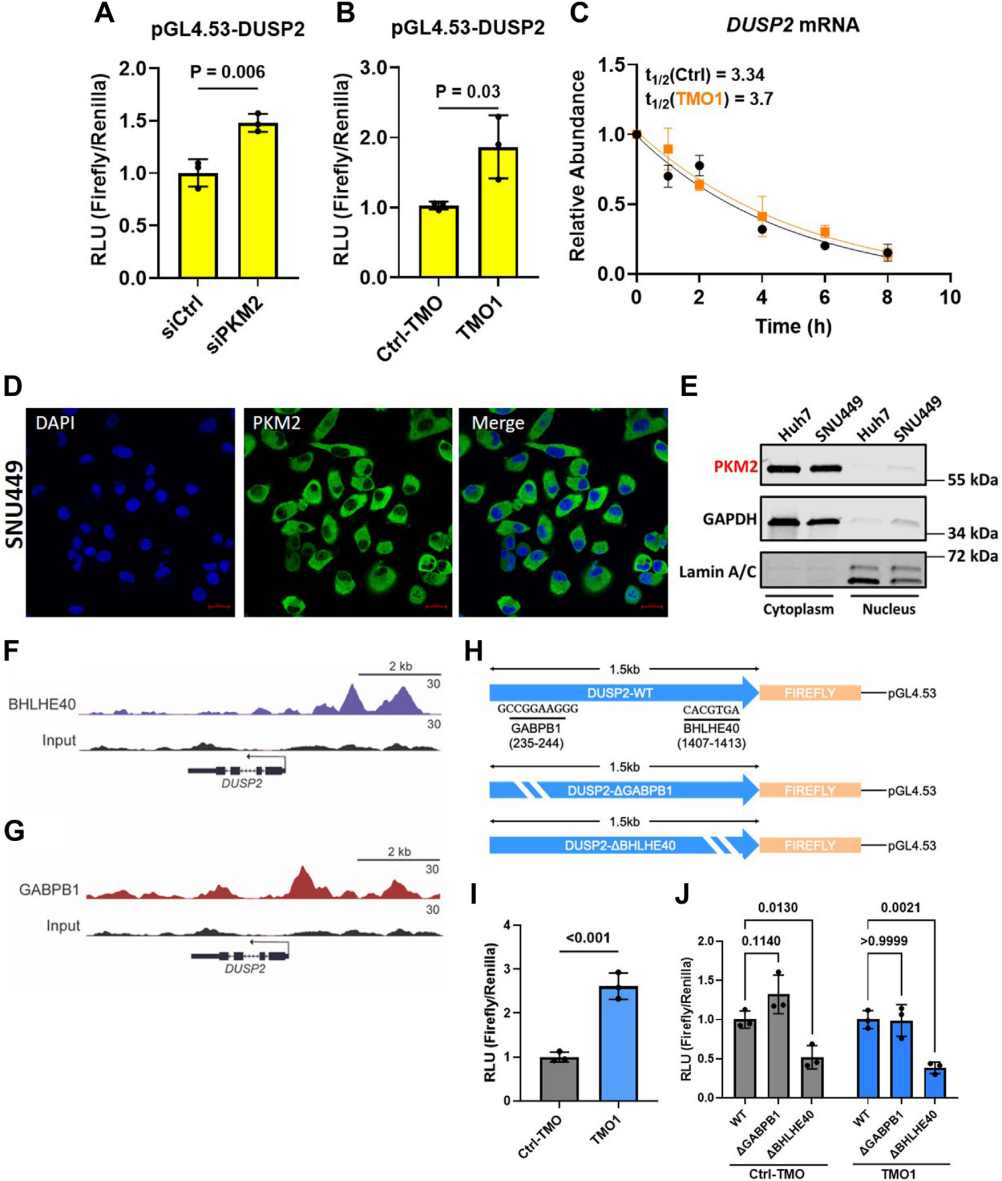
**Downregulation of PKM2 *via* siPKM2 or PKM-TMO induces activation of the *DUSP2* promoter.***A*, firefly luciferase signal from SNU449 cells cotransfected for 48 h with 10 nM siRNA, a firefly luciferase reporter plasmid fused to 1500 bp of DNA sequence upstream from the *DUSP2* transcription start site (pGL4.53-DUSP2), and a Renilla luciferase reporter plasmid with the *PGK* promoter (pRL). The firefly signal was normalized to the Renilla signal. *B*, firefly luciferase signal from SNU449 cells cotransfected for 48 h with 60 nM PKM-TMO, and luciferase plasmids, as described in (*A*). *C*, RT-qPCR of *DUSP2* transcripts in SNU449 cells treated with 60 nM TMO1 or TMO-Ctrl for 48 h, with additional treatment of 10 μg/ml Actinomycin D after 48 h; RNA was extracted at the indicated time points after Act D treatment, and *DUSP2* mRNA was normalized to the *ACTB* mRNA level. Relative expression to cells not treated with Act D is plotted. *D*, representative indirect immunofluorescence images of SNU449 cells stained for PKM2 (*green*) and for nuclei with DAPI (*blue*). Scale bar represents 20 μm. *E*, Western blotting analysis of PKM2 in nuclear and cytoplasmic fractions from Huh7 and SNU449 HCC cell lines. GAPDH and Lamin A/C are indicative of the purity of the cytoplasmic and nuclear fractions, respectively. *F* and *G*, ChIP–seq tracks showing normalized reads of the transcription factors GABPB1 and BHLHE40, along the *DUSP2* promoter. Input DNA is shown below for reference. *H*, diagram of the mutated binding motifs for GABPB1 and BHLHE40 in the pGL4.53-DUSP2 firefly luciferase vector (pGL4.53-DUSP2-ΔGABPB1 and pGL4.53-DUSP2-ΔBHLHE40, respectively). *I*, firefly luciferase signal from SNU449 PKM1-OE cells cotransfected for 48 h with 60 nM TMO1 or TMO-Ctrl, pGL4.53-DUSP2, and pRL. Firefly signal was normalized as in (*B*). *J*, firefly luciferase signal from SNU449 PKM1-OE cells cotransfected for 48 h with 60 nM TMO1 or TMO-Ctrl and either pGL4.53-DUSP2 (WT), pGL4.53-DUSP2-ΔGABPB1, or pGL4.53-DUSP2-ΔBHLHE40, and pRL. The firefly signal was normalized as in (*B*). Bar graphs in (*A* and *B*) and (*I* and *J*) represent the average of three independent biological replicates ± SD, and a two-side *t* test was performed. The line graph in (*C*) represents the average two independent biological replicates ± SD, with lines fitted to points *via* a least-squares fit, and values determined *via* a one-phase decay equation. RLU, relative luciferase units.

We next sought to determine whether decreasing PKM2 with either siPKM2 or PKM-TMO alters DUSP2 expression *via* increased promoter utilization or increased mRNA stability. To this end, we cloned 1500 bp upstream from the human *DUSP2* transcription start site into a firefly luciferase reporter vector. We cotransfected SNU449 cells for 48 h with the firefly plasmid, a renilla luciferase plasmid, and either 60 nM TMO1 or 10 nM of siPKM2. The results showed a significant increase in normalized firefly signal in both siPKM2- and TMO1-treated cells, relative to their respective control (Fig. 4, *A* and *B*). To measure mRNA stability, we transfected SNU449 cells with 60 nM TMO1 or TMO-Ctrl for 48 h and then treated cells with actinomycin D to inhibit transcription, isolated mRNA at 0, 1, 2, 4, 6, and 8 h, and performed RT-qPCR for *DUSP2*. There was no significant difference in the DUSP2 mRNA half-life upon TMO1 treatment, relative to the control (Fig. 4*C*). We conclude that the increase in *DUSP2* mRNA is primarily due to activation of the gene promoter.

Activated ERK1/2 phosphorylates S37 in PKM2, promoting nuclear localization of PKM2 and downstream oncogenic signaling (7). To determine whether PKM2 nuclear localization occurs in HCC cells—which could potentially explain how downregulation of PKM2 results in activation of the *DUSP2* promoter—we performed indirect immunofluorescence analysis of PKM2 in SNU449 cells. We primarily observed PKM2 in the cytoplasm and virtually no nuclear PKM2 (Fig. 4*D*). Moreover, we performed subcellular fractionation to separate the nucleus and cytoplasm of SNU449 and Huh7 cells and carried out Western blotting analysis of PKM2. These results also showed PKM2 primarily in the cytoplasm and almost no PKM2 in the nucleus (Fig. 4*E*). Therefore, although decreased PKM2 leads to increased *DUSP2* expression, it is unlikely that this is due to a nuclear function of PKM2, as opposed to reflecting an indirect effect (see Discussion).

To identify transcription factors that potentially regulate *DUSP2* expression, we analyzed ENCODE ChiP-Seq data from HepG2 cells. We identified GABPB1 and BHLHE40 as potential candidates, given their elevated binding peaks in the region of the *DUSP2* promoter (Fig. 4, *F* and *G*). We next performed motif analysis and identified binding motifs for both GABPB1 and BHLHE40 in our *DUSP2* promoter reporter construct (Fig. 4*H*). We found that deletion of the BHLHE40 motif, but not the GABPB1 motif, significantly reduced *DUSP2* promoter activity, and that treatment with TMO1 was unable to increase the activity of the mutant *DUSP2* promoter (Fig. 4, *I* and *J*). These results suggest that BHLHE40 is involved in the regulation of *DUSP2* expression, together with or downstream of PKM2. Additionally, given that the strongest BHLHE40-binding peak is upstream of our *DUSP2* promoter construct, there may be additional layers of regulation of *DUSP2* expression.

### Upregulation of DUSP2 and DUSP4 following PKM-TMO or siPKM2 treatment correlates with dephosphorylation of ERK1/2 in HCC cells

**Figure 5:**
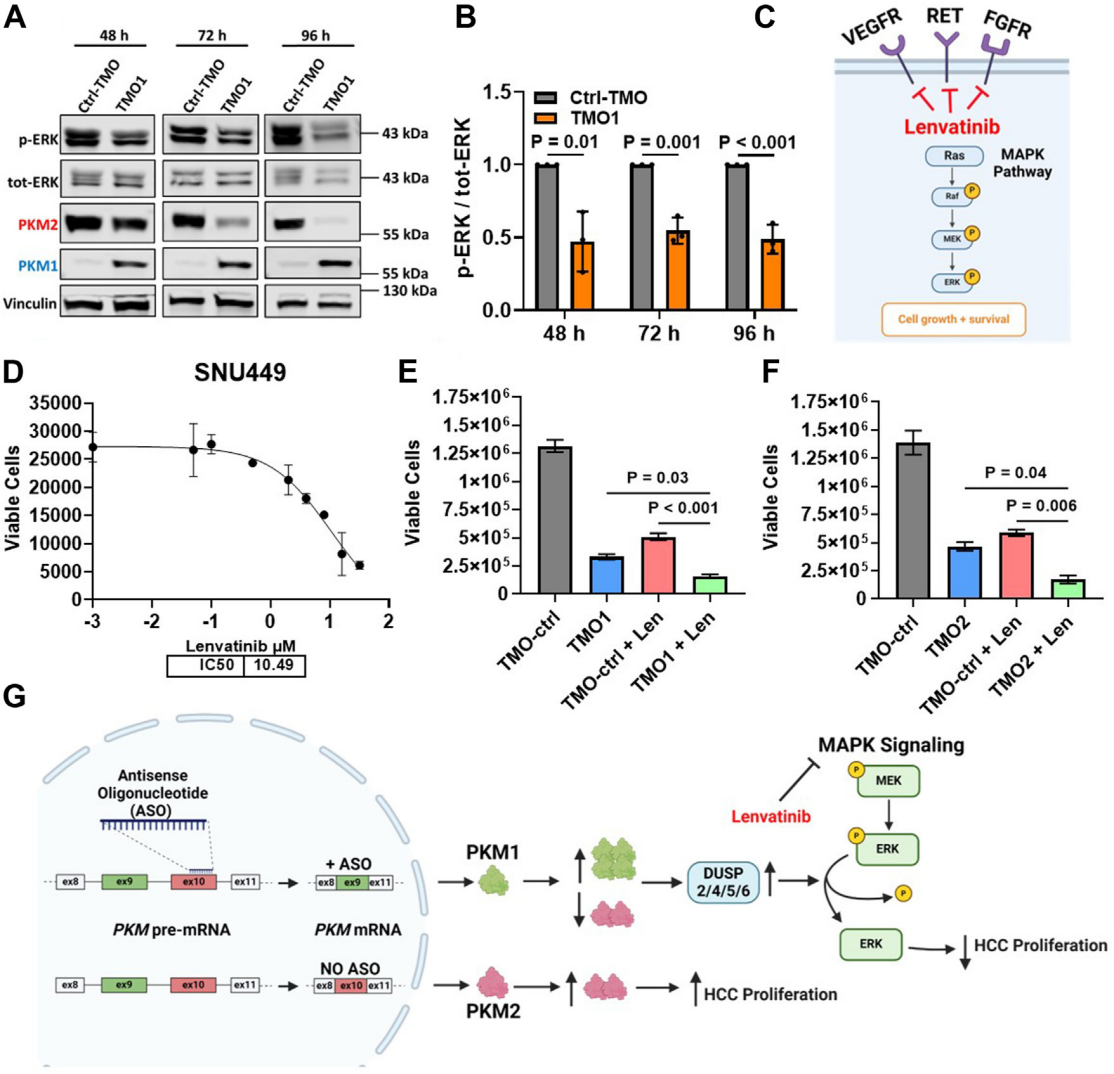
**PKM-TMO treatment in SNU449 cells reduces ERK1/2 phosphorylation and further inhibits cell proliferation when combined with lenvatinib.***A*, representative Western blotting analysis of phosphorylated and unphosphorylated ERK1/2 (p-ERK and tot-ERK, respectively), and PKM isoforms, in SNU449 cells transfected with 60 nM TMO1 for 48, 72, and 96 h. *B*, quantification of the ratio of band intensities for p-ERK and tot-ERK from (*A*); bands were normalized to vinculin, then p-ERK was normalized to tot-ERK, and this ratio was then normalized to Ctrl-TMO. *C*, diagram depicting lenvatinib inhibition of various receptor tyrosine kinase pathways upstream of MAPK. *D*, IC50 curve of SNU449 after 72 h treatment with lenvatinib at the indicated concentrations. Viable cells were counted using flow cytometry. *E*, viable SNU449 cell count after transfection with TMO1 for 24 h, followed by washing and treatment with the IC50 concentration of lenvatinib, relative to 0.3% DMSO control. *F*, viable SNU449 cells counted and treated with TMO2 and lenvatinib as in (*E*). *G*, schematic depicting the effects of treating HCC cells with PKM-ASOs and/or lenvatinib and the resulting downstream alterations in *DUSP* expression and ERK1/2 dephosphorylation. The bar graphs in (*B*, *E*, and *F*) represent the average of three independent biological replicates ± SD. In (*B*), a two-sided *t* test was performed for each time point shown. For (*E* and *F*), one-way ANOVA was performed with Tukey's multiple comparison *post hoc* test. The line graph in (*D*) represents the average of three independent biological replicates ± SD, and the line was fitted to the points with a sigmoidal 4-parameter logistic curve.

In light of the increase we detected in ERK1/2-specific DUSPs as a result of either PKM-TMO or siPKM2 treatment, we next tested whether either treatment resulted in decreased ERK1/2 phosphorylation. To this end, we transfected SNU449 cells with 60 nM TMO1 or TMO-Ctrl for 48, 72, and 96 h and then performed Western blotting analysis of both phosphorylated ERK1/2 (p-ERK) and total ERK (tot-ERK). The results showed a significant 50% reduction in the ratio of p-ERK/tot-ERK for all three time points in the TMO1-treated cells (Fig. 5, *A* and *B*). Additionally, this reduction correlated with increased PKM2-to-PKM1 splice-switching in the TMO1-treated cells for all three time points (Fig. 5*A*). Notably, whereas the extent of PKM splice-switching increased markedly over time, the degree of p-ERK reduction was the same at all time points, which indicates that the effect is saturated within 48 h. Transfection of SNU449 cells for 48 h with 60 nM TMO2 similarly resulted in a significant reduction in the ratio of p-ERK/tot-ERK by 20%, relative to TMO-Ctrl (Fig. S7, *A* and *B*). Finally, transfection of SNU449 cells for 48 h with 5 nM of either si27 or si156 also resulted in a significant reduction in the ratio of p-ERK/tot-ERK by 25%, relative to siCtrl (Fig. S7, *C* and *D*).

### Combined treatment of SNU449 cells with PKM-TMOs and lenvatinib has an additive effect on reducing HCC cell proliferation

The current second-line therapy for treating HCC is with either of two receptor tyrosine kinase (RTK) inhibitors, sorafenib or lenvatinib. Lenvatinib has a relatively high tolerability in HCC patients, compared to sorafenib, as well as a higher objective response rate, which is in part why lenvatinib is more commonly administered to patients (16). Importantly, lenvatinib inhibits multiple RTKs, such as FGFR, RET, and VEGFR, which are all upstream of the MAPK signaling pathway (Fig. 5*C*). Indeed, lenvatinib is well-known to downregulate MAPK signaling, as a result of decreased p-ERK1/2, which leads to reduced HCC growth *in vitro* and *in vivo* (17, 18, 19). Given that our PKM-TMOs also reduce p-ERK, we sought to determine whether combining PKM-TMO treatment with lenvatinib can further reduce HCC cell proliferation. To this end, we established the half-maximal inhibitory concentration (IC50) in SNU449 cells treated with lenvatinib for 72 h (Fig. 5*D*). We then transfected SNU449 cells with 60 nM TMO1 or TMO2 or TMO-Ctrl for 24 h, replenished the media with the IC50 concentration of lenvatinib (10.5 μM), and incubated the cells for an additional 72 h before assessing proliferation with ViaCount. The results showed a detectable decrease in proliferation with TMO1, TMO2, and lenvatinib treatment, relative to their respective controls (Fig. 5, *E* and *F*). The combination treatment with lenvatinib and TMO1, or lenvatinib and TMO2, resulted in a significantly greater reduction in proliferation relative to either treatment alone, which demonstrates that PKM-TMOs increase the sensitivity of HCC cells to lenvatinib treatment (Fig. 5, *E-G*).

## Discussion

PKM-ASOs induce simultaneous upregulation of PKM1 and downregulation of PKM2, decreasing HCC cell proliferation *in vitro* within days of treatment (10). Moreover, subcutaneous delivery of PKM-ASOs to immunocompromised mice harboring human HCC orthotopic xenografts, or to immunocompetent mice harboring *de novo* HCC, limits tumor growth relative to control-treated mice (10). PKM1 is a known tumor-suppressor in various cancer contexts, and decreased PKM2 expression in HCC is correlated with increased overall survival, recurrent-free survival, and overall prognosis (10, 20, 21, 22). In this study, we sought to deconvolve the PKM1- and PKM2-specific effects of PKM-ASO treatment in HCC.

We observed that either dox-inducible overexpression of PKM1 or small-molecule activation of PKM2 significantly increased PK activity in HCC cells within days, but surprisingly did not alter HCC proliferation. On the other hand, treating HCC cells with PKM-ASOs significantly reduced proliferation within days. Therefore, the effect on proliferation is likely not due to the acute increase in PKM1 expression or in PK activity, which argues against a tumor-suppressive role for PKM1 in HCC. We previously observed that either downregulation of PKM2, or transfection with *PKM* splice-switching ASOs, induced apoptosis in glioma cell lines and that overexpression of exogenous PKM1 had no effect (8). These results further suggest that acute downregulation of PKM2, but not overexpression of PKM1, is detrimental to cancer-cell proliferation (8). However, we also previously observed that HCC cells that stably overexpress PKM1 form fewer colonies in soft agar when incubated for more than 3 weeks (10). Therefore, we speculate that longer exposure to PKM1 overexpression, or treatment with PKM-ASOs, may rewire metabolism and induce metabolic vulnerabilities that limit HCC progression *in vivo*.

Importantly, we observed that treating three different liver-cancer cell lines with either of two different PKM2-targeting siRNAs resulted in a dose-dependent reduction in proliferation within 5 days. Given the similarities in growth reduction between siPKM2 and PKM-ASO treatment, these results suggest that the acute reduction in HCC proliferation following PKM-ASO treatment is primarily due to downregulation of PKM2. This brings into question whether ASO-based PKM splice-switching is advantageous in the HCC context and whether developing PKM-ASOs that selectively reduce PKM2 mRNA would be a more logical treatment strategy. To this end, we could utilize an ASO “gapmer” approach, which is based on ASOs that can induce RNase H–mediated cleavage and degradation of PKM2 mRNA (23). Although this approach is similar to siPKM2, ASOs have the advantage of not requiring lipid nanoparticle formulation. On the other hand, uniformly modified ASOs are more stable than gapmer ASOs, and because they do not elicit RNA cleavage by RNase H, they are expected to have fewer off-target effects.

It is not intuitive why knocking down a glycolytic enzyme with severely diminished activity significantly reduces HCC cell proliferation. Therefore, to better understand the impact of decreased PKM2 expression in HCC, we performed transcriptomic analysis on SNU449 HCC cells treated with either siPKM2 or PKM-ASOs and identified overlapping alterations in gene expression. The results, together with RT-qPCR validation, showed significantly increased expression of various DUSPs (DUSP2, DUSP4, DUSP5, and DUSP6). Moreover, we observed similar upregulation of *DUSP2* mRNA in SNU398 and Huh7 HCC cell lines treated with PKM-ASO. Downstream target validation confirmed detectable increases in DUSP2 and DUSP4 protein levels following treatment of SNU449 cells with either siPKM2 of PKM-ASO.

Notably, DUSP2 and DUSP4 both dephosphorylate ERK1/2, which is a downstream signaling node in the pro-proliferative component of the MAPK signaling pathway (15). Indeed, siRNA suppression of DUSP2 and DUSP4 in various cancers is known to increase tumor progression and chemoresistance (15, 24, 25, 26). Given that we were unable to detect PKM2 in the nucleus, we suspect that the effect of PKM2 downregulation on increased DUSP expression may be the result of PKM2 sequestering relevant proteins in the cytoplasm. For example, PKM2 is known to sequester β-catenin in the cytoplasm, which then translocates to the nucleus upon downregulation of PKM2 (27).

We also found that mutating a putative BHLHE40 transcription factor–binding motif in the *DUSP2* promoter reduced *DUSP2* activation and prevented PKM-ASO induction of DUSP2. Indeed, a recent report showed that *Bhlhe40* expression is strongly correlated with *Dusp2* expression in mouse intratumoral T cells (28). Although the regulatory interplay between BHLHE40 and PKM2 remains to be explored, both proteins have been implicated in the hypoxia response pathway: PKM2 aids in the transactivation of HIF-1, whereas hypoxia upregulates BHLHE40 expression (29, 30). Interestingly, it is also known that hypoxia suppresses *DUSP2* expression in various cancers (24). Therefore, we speculate that there is a complex interplay between PKM2, BHLHE40, and DUSPs, which share a common signaling node in the hypoxia response pathway.

As both siPKM2 and PKM-ASO treatment resulted in increased expression of various ERK1/2-specific phosphatases, especially DUSP2, we sought to determine whether this effect correlated with a decrease in ERK1/2 phosphorylation. Indeed, we observed that treatment with either PKM-ASOs or siPKM2s resulted in decreased phosphorylation of ERK1/2. PKM2 was reported to act as a protein kinase that can directly interact with and phosphorylate ERK1/2 on T202, in addition to phosphorylating other proteins (31). However, another study that attempted to reproduce these findings failed to obtain any evidence for PKM2 acting as a protein kinase, and the authors suggested that the original findings were confounded by contamination with other protein kinases (32). We speculate that another possibility is that increased expression of PKM2 leads to suppression of ERK1/2-specific phosphatases, namely the various DUSPs identified in this study, which would result in accumulation of phosphorylated ERK1/2 that could be mistaken for PKM2-specific phosphorylation of ERK1/2.

Notably, PKM2 is also a direct target of ERK1/2 in various cancers, with phosphorylation of PKM2 at S37 causing nuclear translocation and downstream activation of oncogenes (1, 7). However, in the present study, we primarily detected PKM2 in the cytoplasm of HCC cells, so it is unlikely that nuclear PKM2 mediates the decreased proliferation we observed with either siPKM2 or PKM-ASO treatment. Still, we cannot rule out the possibility that a very small fraction of PKM2 translocates to the nucleus to affect transcription of target genes. If that were the case, however, DUSP activation upon downregulation of PKM2 should decrease ERK1/2 phosphorylation, in turn decreasing PKM2 phosphorylation and nuclear translocation. Irrespective of the precise mechanism, considering that phosphorylation of ERK1/2 induces downstream activation of various pro-proliferative and pro-survival genes in cancer (33), our observation that decreased PKM2 correlates with an increase in DUSPs and a decrease in ERK1/2 phosphorylation likely accounts for the reduction in HCC cell proliferation following either siPKM2 or PKM-ASO treatment.

Our analysis of The Cancer Genome Atlas data surprisingly showed a direct, rather than an inverse correlation between PKM2 expression and DUSP expression in HCC tumors. Our interpretation of this apparent discrepancy is that the cell-culture experiments reflect an acute response to PKM2 downregulation, whereas the expression data from HCC tumors presumably reflect selection for sustained tumor growth, including metabolic rewiring and other adaptations.

Finally, we observed that combined treatment with lenvatinib and either PKM-ASO resulted in a greater reduction in SNU449 cell growth than either treatment alone. Notably, another report showed that treating HCC cells with miR-374b indirectly reduces PKM2 levels *via* downregulation of the PKM1 splicing repressor hnRNPA1, which resensitizes HCC tumors to the RTK inhibitor sorafenib—a less commonly used second-line therapy for HCC (34, 35, 36, 37). Considering that lenvatinib is the preferred second-line therapy for HCC, it will be of interest to conduct *in vivo* studies that compare the potential tumor-suppressive effects of combining PKM-ASOs with lenvatinib to either treatment alone. Moreover, first-line HCC therapy consists of atezolizumab (immune checkpoint inhibitor) plus bevacizumab (angiogenesis inhibitor), which is contraindicated in patients with autoimmune disease and/or untreated varices (37). Therefore, bolstering second-line HCC therapies with PKM-ASOs could provide further benefit to these vulnerable patients.

## Experimental procedures

### Cell culture

Cells were used within 10 passages for all experiments and were confirmed to be mycoplasma-free routinely during the course of the study, using a Lonza MycoAlert kit. Cells were cultured at 37 °C and 5% CO2. HepG2 (human hepatoblastoma), SNU449, and SNU398 (human HCC) cells were obtained directly from ATCC and cultured in RPMI 1640 (Corning) supplemented with 10% fetal bovine serum and 1% penicillin/streptomycin (P/S). Huh7 (human HCC) cells were obtained from the Japanese Collection of Research Bioresources Cell Bank and validated *via* STR profiling (University of Arizona Genetics Core) and cultured in RPMI 1640 supplemented with 10% fetal bovine serum and 1% P/S.

### ASOs and siRNAs

All ASOs used in this study were mixed-chemistry oligonucleotides (Table S2). cEt ASO chemistry (38) and the cEt ASOs used in this study (10) were described previously. TMO synthesis and purification were as described (14). All ASOs had uniform phosphorothioate or thiophosphoramidate morpholino backbones and 5-methylcytosine. We dissolved the ASOs in water and stored them at −20 °C. ASO concentration was determined with a Nanodrop spectrophotometer. ON-TARGETplus siRNAs (Table S3) were purchased from Horizon Discovery.

### Delivery of ASOs and siRNAs

For transfection with either siRNA or ASO in a 6-well plate, 7.5 μl lipofectamine 3000 (Thermo Fisher Scientific) was mixed with 250 μl opti-MEM (Thermo Fisher Scientific), then mixed with 250 μl opti-MEM containing 10× of the final ASO or siRNA concentration, and incubated for 20 min. Five hundred microliters of the mix was then added dropwise to 2 ml of culture medium without 1% P/S. These reaction volumes were scaled according to the cell-culture surface area.

### Plasmids and stable cell lines

The human *DUSP2* promoter sequence (gene ID: 1844) was synthesized by GenScript and spans 1500 bp upstream from the transcription start site and flanking 5′ KpnI and 3′ NcoI restriction enzyme sites. The DNA fragment was cloned into the pGL4.53 firefly luciferase vector (Promega) *via* restriction sites. Transcription factor–binding site deletion mutants were generated using a Q5 Site-Directed Mutagenesis Kit (NEB) with nonoverlapping primers flanking each binding motif. The N-terminal flag-tagged human PKM1 cDNA (NM_182471.4), containing flanking attL regions for LR Gateway Cloning, was synthesized by Genscript and cloned into the pCW57.1 vector, which was a gift from David Root (Addgene #41393). The *PGK* promoter was swapped with a CMV promoter using Gibson Assembly (NEB). Lentiviruses were produced in HEK293T/17 cells by cotransfecting viral constructs with psPAX2 and vesicular stomatitis virus G glycoprotein. Viral supernatant was collected 48 to 72 h post-transfection and concentrated with a Lenti-X concentrator (Takara Bio) according to the manufacturer's instructions. Cells were infected with concentrated lentiviral particles overnight, in the presence of 8 μg/ml polybrene (Sigma), and selected using puromycin (Sigma) for at least 2 weeks to generate stable cell lines. For selection, HepG2 and SNU398 cells were treated with 1.5 μg/ml puromycin and SNU449 cells with 10 μg/ml puromycin.

### RT-qPCR

Cells were briefly washed in ice-cold PBS, then 1 ml Trizol (Thermo Fisher Scientific) was added directly to 10^6^ cells. RNA was isolated using the Direct-zol RNA Miniprep kit (Zymo Research) and reverse-transcribed with ImProm-II reverse transcriptase (Promega) using oligo-dT primers. PowerUp SYBR Green master mix was used to prepare RT-qPCR reactions (Applied Biosystems), which were analyzed on a QuantStudio 6 Flex Real-Time PCR system (Thermo Fisher Scientific). Fold changes were calculated using the ΔΔCt method. RT-qPCR primers are listed in Table S4.

### mRNA stability

Cells (5 × 10^4^) were seeded in 12-well plates overnight, then transfected with 60 nM TMO1 or TMO-Ctrl for 48 h. Culture medium was then replaced with medium containing 10 μg/ml actinomycin D (Sigma), and cells were incubated for 0, 1, 2, 4, 6, and 8 h. For each time point, cells were briefly washed in 1 ml ice-cold PBS, and 500 μl Trizol was added directly to the cells for 5 min. Samples were then transferred to 1.5 ml Eppendorf tubes and stored at −80 °C. RNA isolation and RT-qPCR were then performed as described above. The ΔΔCt values were calculated based on the 0 h time point. Half-life values were calculated in Prism 10 using the one-phase decay least-squares-fit equation.

### Western blotting

Cells were washed in ice-cold PBS, then radioimmunoprecipitation assay buffer containing 1× protease inhibitor cocktail (Roche) was added. To detect phosphorylated proteins, Halt phosphatase inhibitor cocktail (Thermo Fisher Scientific) was added to lysis buffer at a 1:50 dilution. Cells were then scraped and collected into 1.5 ml Eppendorf tubes and incubated on ice for 20 min. Lysates were then cleared by centrifugation and protein was quantified *via* Bradford assay (Bio-Rad) and bovine serum albumin (BSA) standard curve. Twenty five micrograms of protein lysate was then combined with 10 mM DTT and 1× Laemmli buffer and boiled for 5 min at 95 °C. Protein lysates were then separated *via* SDS-PAGE and transferred onto a nitrocellulose membrane. The membrane was blocked with 5% BSA in Tris-buffered saline with 0.1% Tween 20 detergent (TBST) and incubated overnight at 4 °C with primary antibody diluted 1:1000. The membrane was then incubated with goat anti-mouse or goat anti-rabbit Li-Cor IRDye 800 (green) or 680 (red) secondary antibodies (1:10,000; LI-COR Biosciences) for 1 h at room temperature. Protein bands were visualized on an Odyssey imaging system (Li-Cor) and quantified using ImageStudio and ImageJ. Primary antibodies used in this study are listed in Table S5.

### Immunofluorescence staining

SNU449 cells were seeded to 75% confluency in a 24-well glass plate and incubated for 24 h. Cells were then washed with PBS and fixed with 4% paraformaldehyde for 10 min. Cells were then permeabilized with 0.1% Triton X-100 in PBS and incubated for 15 min. Cells were then blocked with 2% BSA + 5% goat serum in PBS for 60 min. Primary PKM2 antibody was diluted to 2.5 μg/ml in 0.1% BSA + 1% goat serum and incubated with the cells overnight at 4 °C. Cells were then washed in PBS and incubated with fluorescent-dye-labeled secondary antibody diluted 1:10,000 in 0.1% BSA + 1% goat serum for 45 min at room temperature. DAPI was then added to the cells for 2 min, followed by washing with PBS. Images were captured on a Zeiss LSM780 confocal laser-scanning microscope.

### Subcellular fractionation

Nuclear and cytoplasmic fractions were prepared from 5 × 10^6^ cells, according to the Subcellular Protein Fractionation Kit manufacturer's instructions (Thermo Fisher Scientific). Lysates were then prepared for Western blotting analysis as described above.

### Cell counting with flow cytometry

For all proliferation assays, we utilized a benchtop flow cytometer Guava EasyCyte HT-BG (Cytek) paired with automated cell counting using ViaCount (Cytek). Cell processing and counting were conducted as previously described (10).

### Lenvatinib IC50 and combined treatment with PKM-TMO

Lenvatinib (Selleckchem) was initially dissolved to 100 mM in DMSO and stored at −80 °C. Cells (7.5 × 10^3^) were seeded in a 96-well plate overnight and treated the following day with the indicated concentrations of lenvatinib. All dilutions of lenvatinib were diluted in DMSO to a final concentration of 0.3%. Seventy two hours post-treatment, the cells were processed for cell counting with ViaCount as described above. Viable cell counts and their corresponding log-transformed concentrations were then plotted in Prism and fitted with a sigmoidal 4-parameter logistic curve (4PL). The IC50 was then calculated in Prism using the equation for “log(inhibitor) *versus* response – variable slope (four parameters)”. For cotreatment with lenvatinib and PKM-TMO, cells (2.5 × 10^5^) were seeded in a 6-well plate overnight, then transfected with 30 nM of either TMO1, TMO2, or TMO-Ctrl for 24 h. The medium was then replaced with fresh medium containing the IC50 concentration for lenvatinib (10.5 μM), and the cells were incubated for an additional 72 h, followed by cell counting with ViaCount.

### *DUSP2* promoter activity assay

SNU449 cells were seeded in a 12-well plate and cotransfected with 10 nM siPKM2 or 60 nM TMO1, pRL renilla luciferase plasmid (Promega), and pGL4.53-DUSP2 (or pGL4.53-DUSP2-ΔGABPB1 or pGL4.53-DUSP2-ΔBHLHE40) *via* lipofectamine 3000 (Invitrogen) for 48 h. Luciferase assays were performed using the Dual-Luciferase Reporter Assay System (Promega) according to the manufacturer's instructions. Luciferase activity was measured using a SpectraMax i3 plate reader (Molecular Devices).

### Identification of transcription factors bound to the *DUSP2* promoter

ChIP-seq peaks for 132 transcription factors in HepG2 from ENCODE were initially loaded onto the UCSC genome browser (GRCh38) for visualization at the *DUSP2* locus (39). For each factor with a called peak at the *DUSP2* locus, fastq files were accessed from GSE104247 for processing. Raw reads from fastq files were aligned to the reference human genome assembly hg38, using Bowtie2 with defaults (40). Peaks were called using MACS2 and annotated using the annotatePeaks from HOMER (41, 42). The DeepTools bamCompare tool was used to generate bigwig files for visualization on the UCSC genome browser. After validation that each factor binds to the *DUSP2* locus, the *DUSP2* promoter was subjected to motif analysis using FIMO within the MEME suite to identify specific transcription factor–binding motifs. Each motif identified by FIMO was individually deleted on the DUSP2 promoter plasmid, and the luciferase reporter assay was repeated.

### PK activity assay

PK activity was measured with a Pyruvate Kinase Activity Colorimetric/Fluorometric Assay Kit (BioVision) in accordance with the manufacturer's instructions. Cells were seeded at ∼30% confluency and treated with either TEPP-46 or dox at the indicated concentrations and time points for 5 days. Cells were then lysed in 100 μl of assay buffer and processed according to the manufacturer's instructions. Ten microliters of lysate was used per reaction. Optical density at 570 nm was measured at room temperature using a SpectraMax i3 plate reader (Molecular Devices) from 0 to 45 min at 30-s interval time points. A duplicate plate was then counted with ViaCount, and the cell numbers were used for normalization.

### Bulk RNA-seq library preparation

SNU449-PKM1OE cells (2.5 × 10^5^) were seeded in 6-well plates and treated as indicated for 48 h. RNA isolation was performed using a Direct-zol RNA Miniprep kit (Zymo Research). RNA concentration and quality were determined *via* Qubit and Tape Station assessment (Agilent), which detected an RNA Integrity Number (RIN) greater than 9.5 for all samples. RNA-seq libraries were then prepared with Kapa mRNA Hyper Prep kit (Roche). Samples were then pooled, and the pool was sequenced *via* paired-end (101 bp) sequencing with dual-indexed reads on a P3 chip using an Illumina NextSeq2000. Each sample was sequenced to an average depth of ∼27 million reads.

### Bulk RNA-seq analysis and quality control

Bulk RNA-seq analyses were implemented and integrated using the “CodeSpringLab” platform developed by the CSHL Bioinformatics core, downloaded from https://github.com/RadUtama/CodeSpringLab.git. Quality control of raw sequencing files (fastq) was implemented using FastQC v0.11.8 and FastQ Screen v0.15.2 with default parameters.

### Alignment and gene quantification

Sequence alignment was performed using STAR v2.7.10a with parameters set to “--outFilterMismatchNmax-2,” “--outFilterMultimapNmax-2,” “--outSAMtype BAM SortedByCoordinate,” “--outSAMunmapped None,” “--outSAMstrandField None.” The human genome reference (FASTA assembly and GTF annotation) was extracted from GENCODE GRCh38.p13 v42. Gene quantification was performed using featureCounts from Subread v2.0.2 with parameters set to “-p --countReadPairs,” “-t exon,” “-s 0,” “-Q 12,” “-C –minOverlap 1.”

### Differential gene analysis

Differential analysis was performed using DESeq2 v1.36 and log-fold shrinkage “apeglm” package, with parameters set to a minimum of 10 read counts for each gene summed over all samples. DEGs with a false discovery less than 0.05 were then filtered by log2FoldChange greater than 0.585 or less than −0.585. The filtered gene lists were then entered into https://bioinformatics.psb.ugent.be/webtools/Venn/ to identify both unique and shared DEGs.

### Pathway analysis

Each list of DEGs was uploaded to DAVID (43) and converted to HGNC gene symbols *via* the gene conversion tool. Functional annotation clustering with medium stringency was performed on Gene Ontology, Kyoto Encyclopedia of genes and genomes, and Biocarta databases (44, 45, 46). To correct for multiple hypothesis testing, the Benjamini–Hochberg method was applied to the original *p*-values that were identified for each term, and only terms with an adjusted *p*-value ≤ 0.05 were kept for further investigation.

### Statistical analysis

Statistical analyses and data visualization were performed using Prism 10 (GraphPad Software) unless stated otherwise. For comparisons between two groups, a two-tailed unpaired *t* test was performed. One-way ANOVA with Tukey multiple comparison *post hoc* test was performed when comparing three or more groups. Statistical significance is indicated by ∗*p* ≤ 0.05; ∗∗*p* ≤ 0.01; ∗∗∗*p* ≤ 0.001. “NS” indicates no significance (*p* > 0.05). Data are expressed as mean ± SD.

## Data availability

The TCGA (The Cancer Genome Atlas) data are publicly accessible through the NCI Genomic Data Commons (GDC) (https://gdc.cancer.gov/). The source code for RNA-seq analysis is available at GitHub *via* https://github.com/RadUtama/CodeSpringLab.git. All data are available in the main text or in the supporting information section.

## Supporting information

This article contains supporting information.

## Conflict of interest

A. R. K. discloses the following commercial relationships, unrelated to the present work: Stoke Therapeutics (Co-Founder, Director and Chair of SAB); SABs of Skyhawk Therapeutics, Envisagenics, and Autoimmunity BioSolutions; and Consultant for Biogen, SEED Therapeutics, Crucible Therapeutics, Cajal Neuroscience, and Collage Bio. In addition, A. R. K. is an inventor on an issued patent covering PKM ASOs. M. H. C. discloses the following commercial relationships, unrelated to the present work: Board of Directors and Co-Founder of ProGenis, SynGenis, and LincSwitch; Board of Directors of Vesicle Therapeutics; Consultant for AmideBio. C. R. V. has received consulting fees from Flare Therapeutics, Roivant Sciences, and C4 Therapeutics; has served on the advisory boards of KSQ Therapeutics, Syros Pharmaceuticals, and Treeline Biosciences; has received research funding from Boehringer-Ingelheim and Treeline Biosciences; and owns a stock option from Treeline Biosciences. All other authors declare that they have no conflicts of interests with the contents of this article.
