# Supplementary material for: PKM splice-switching ASOs induce upregulation of dual-specificity phosphatases and dephosphorylation of ERK1/2 in hepatocellular carcinoma
Source: J Biol Chem. 2025 Feb 22;301(4):108345. doi: 10.1016/j.jbc.2025.108345 (PMC11982463; doi:10.1016/j.jbc.2025.108345)
Supplement: Supporting information [file mmc1.docx]

SUPPORTING INFORMATION

***PKM* splice-switching ASOs induce upregulation of dual-specificity phosphatases and dephosphorylation of ERK1/2 in hepatocellular carcinoma**

Dillon M. Voss^1,2^, Alexander J. Kral^1,3^, GeunYoung Sim^1^, Raditya Utama^1^, Kuan-Ting Lin^1^, Chris Cizmeciyan^1^, Balazs Schafer^4^, Patrick J. Cunniff^1^, Christopher R. Vakoc^1^, Marvin H. Caruthers^4^, Lopa Mishra^5^, and Adrian R. Krainer^1^

**Affiliations:**

1. Cold Spring Harbor Laboratory, Cold Spring Harbor, New York, USA
2. Renaissance School of Medicine, Stony Brook University, Stony Brook, New York, USA
3. Department of Microbiology and Immunology, Renaissance School of Medicine, Stony Brook University, Stony Brook, New York, USA
4. Department of Biochemistry, University of Colorado, Boulder, Colorado, USA.
5. The Institute for Bioelectronic Medicine, The Feinstein Institutes for Medical Research, Division of Gastroenterology and Hepatology, Northwell Health, New York, USA

For correspondence: Adrian R. Krainer, Krainer@cshl.edu

**Running title:** PKM-ASO treatment upregulates DUSP2 in HCC

**Keywords:** Cancer, Antisense RNA, pyruvate kinase, hepatocellular carcinoma, dual‐specificity phosphoprotein phosphatase

**Table of Contents**

Supplemental Tables S1-S5

Supplemental Figure S1

Supplemental Figure S2

Supplemental Figure S3

Supplemental Figure S4

Supplemental Figure S5

Supplemental Figure S6

Supplemental Figure S7

**Table S1. Subcellular localization of proteins encoded by the 264 DEGs from RNA-seq**

**
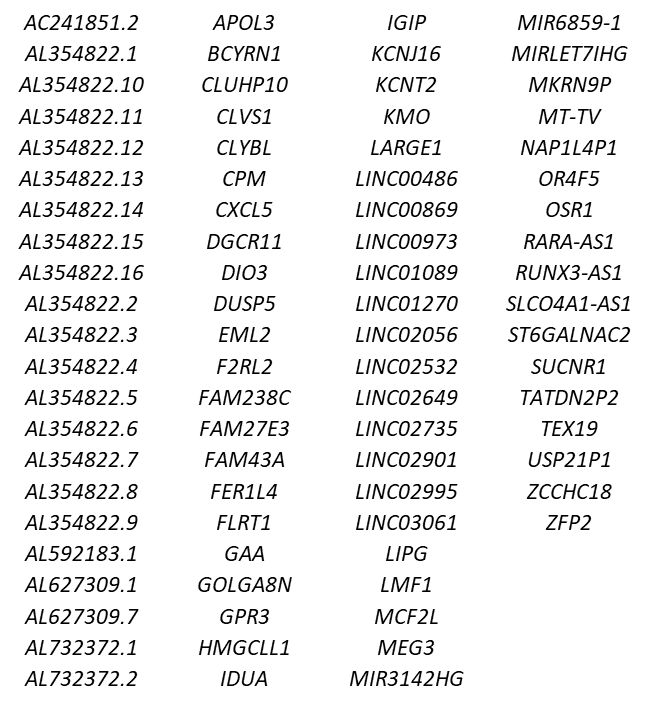
**

**HPA subcellular location undetermined*

**S** = secreted, **C** = cytosol, **NS** = nuclear speckle, **Nu** = nucleus, **Nc** = nucleoli, **NM** = nuclear membrane, **M** = mitochondria, **PM** = plasma membrane, **Micro** = microtubule, **Golgi** = golgi apparatus, **Csome** = centrosome, **Cmere** = centromere, **ER** = endoplasmic reticulum, **CJ** = cell junction, **F** = flagellar centriole, **PC** = primary cilium. Localization determined using The Human Protein Atlas (HPA), [http://www.proteinatlas.org](http://www.proteinatlas.org/).

**Table S2. ASOs**

| **ASO name** | **Sequence 5’ – 3’** | **Chemistry** |
| --- | --- | --- |
| cEt-ASO1 | AGGCGGCGGAGTTCCTCA | kkddkddkddkddkddkk |
| TMO1 | AGGCGGCGGAGTTCCTCA | pppppppppppppppppd |
| TMO2 | GTGAGGACGATTATGGCC | pppppppppppppppppd |
| TMO-Ctrl | GTTGCATACGCGAGGCGC | pppppppppppppppppd |

*Base modification: k = constrained ethyl (cEt) base modification; p = thiophosphoramidate morpholino (TMO); d = DNA base; all ASOs have uniform phosphorothioate or thiophosphoramidate morpholino backbone and 5-methyl-C modifications.

**Table S3. siRNAs**

| **siRNA** | **Sense 5’-3’** | **Antisense 5’-3’** |
| --- | --- | --- |
| siCtrl | AGGCAGAGGCUGCCAUCUAUU | UAGAUGGCAGCCUCUGCCUUU |
| si27 | CCAUAAUCGUCCUCACCAAUU | UUGGUGAGGACGAUUAUGGUU |
| si156 | CUUACGCUGAGUACUUCGAUU | UCGAAGUACUCAGCGUAAGUU |

**Table S4. Primers**

| **Primer** | **Forward 5’-3’** | **Reverse 5’-3’** |
| --- | --- | --- |
| ACTB | GGACTTCGAGCAAGAGATGG | AGCACTGTGTTGGCGTACAG |
| DUSP2 | GGGCTCCTGTCTACGACCA | GCAGGTCTGACGAGTGACTG |
| DUSP4 | GGCGGCTATGAGAGGTTTTCC | TGGTCGTGTAGTGGGGTCC |
| DUSP5 | TGTCGTCCTCACCTCGCTA | GGGCTCTCTCACTCTCAATCTTC |
| DUSP6 | GAAATGGCGATCAGCAAGACG | CGACGACTCGTATAGCTCCTG |

**Table S5. Antibodies**

| **Antibody** | **Source** | **Catalog #** |
| --- | --- | --- |
| PKM1 (D30G6) Rabbit mAb | Cell Signaling | 7067S |
| PKM2 (D78A4) Rabbit mAb | Cell Signaling | 4053S |
| DUSP2 (PAC-1) Mouse mAb | Santa Cruz | sc-32776 |
| DUSP4 (MKP2) Rabbit mAb | Cell Signaling | 5149S |
| DUSP5 (H-9) Mouse mAb | Santa Cruz | sc-393801 |
| DUSP6 Rabbit mAb | Abcam | Ab76310 |
| P-p44/42 MAPK (ERK1/2) Rabbit mAb | Cell Signaling | 4370S |
| p44/42 MAPK (Erk1/2) (137F5) Rabbit mAb | Cell Signaling | 4695S |


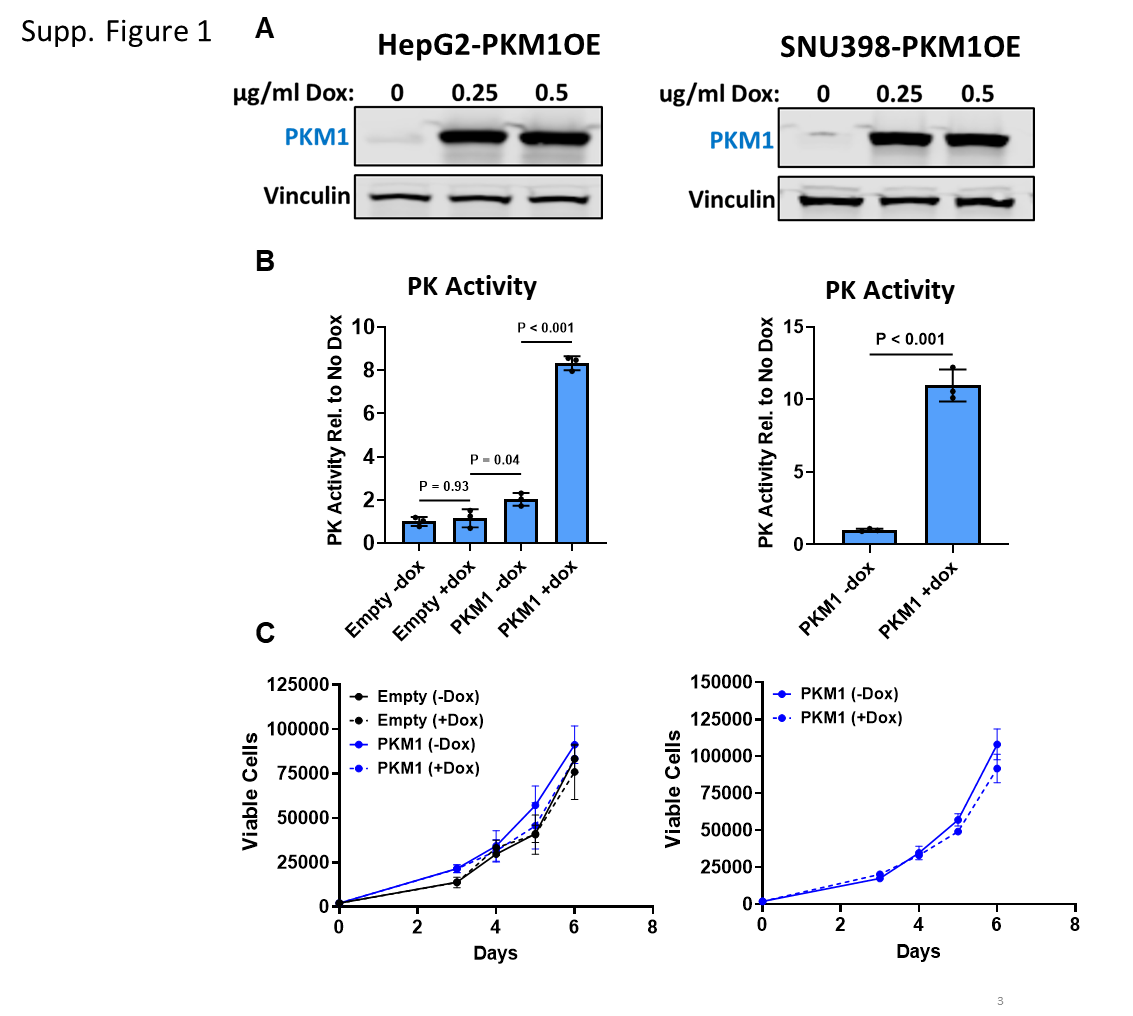


**Supplemental Figure 1. Dox-inducible expression of Flag-PKM1 increases PK activity, but does not alter cell proliferation.** (A) Western blotting analysis of PKM1 protein levels in PKM1-OE liver-cancer cell lines (HepG2, left and SNU398, right) after treatment with the indicated concentration of dox for 5 d. Cells were treated with dox on days 0, 2, and 4. (B) PK activity in PKM1-OE cells treated with 0.25 µg/mL dox on days 0, 2, and 4. PK activity was quantified on day 5. (C) Viable cells were counted using ViaCount with flow cytometry. Cells were treated as in (B). One-way ANOVA was performed with Tukey’s multiple comparison post-hoc test for data on the left, and a two-sided *t*-test was performed for data on the right. The bar and line graphs in (B) and (C) represent the average of three independent biological replicates ± SD. There was no significant change detected between treatment groups in (C).


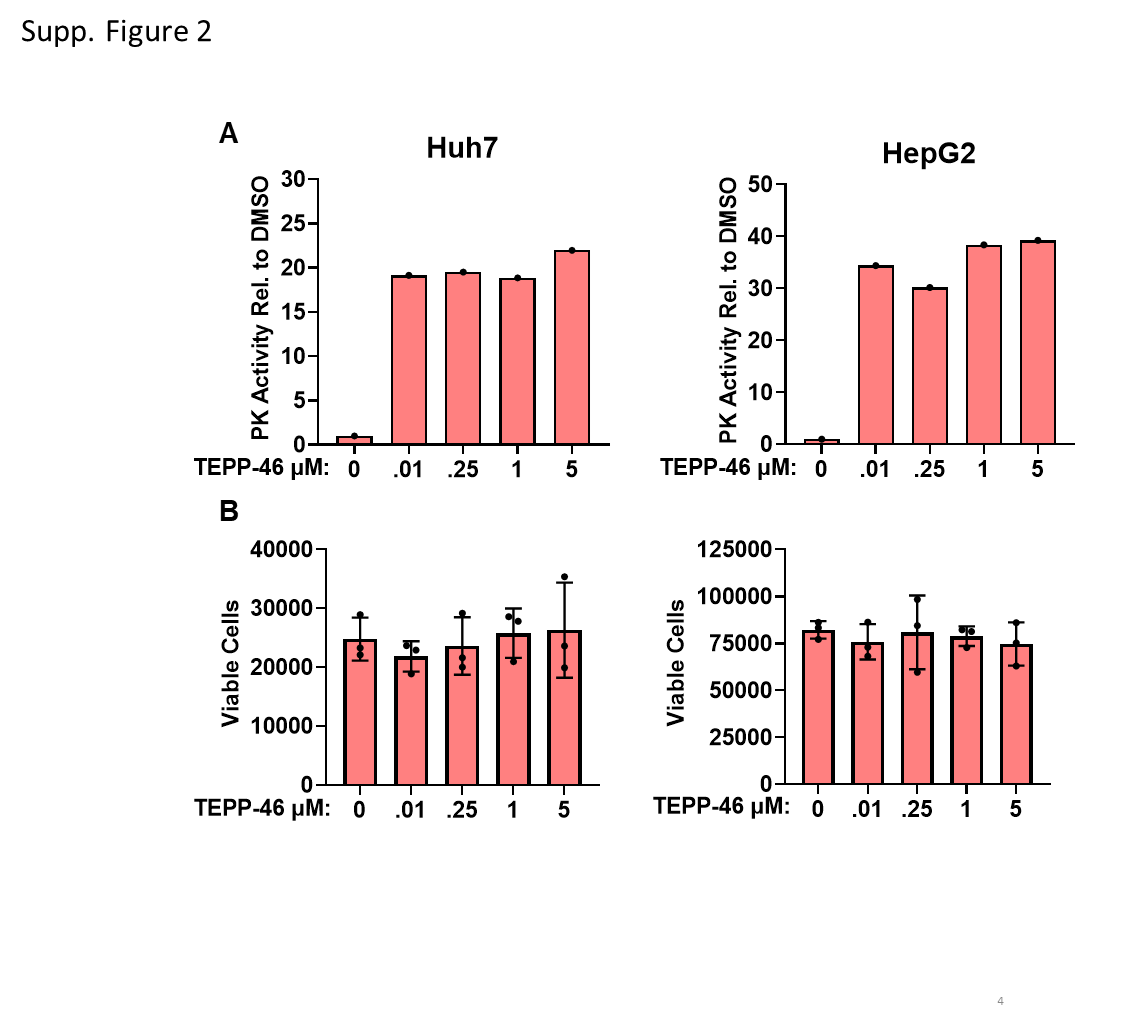
**Supplemental Figure 2.** **Small-molecule activation of PKM2 with TEPP-46 increases PK activity, but does not alter cell proliferation.** (A) PK activity on day 5 in Huh7 (left) and HepG2 (right) cells treated with TEPP-46 on days 0, 2, and 4, at the indicated concentrations. (B) Viable cells were counted on day 5 using ViaCount and flow cytometry. Cells were treated with TEPP-46 as in (A). The bar graphs in (A) represent single measurements at each drug concentration. The bar graphs in (B) represent the average of three independent biological replicates ± SD, and one-way ANOVA was performed with Tukey’s multiple comparison post-hoc test, which showed no statistically significant differences between any of the conditions.


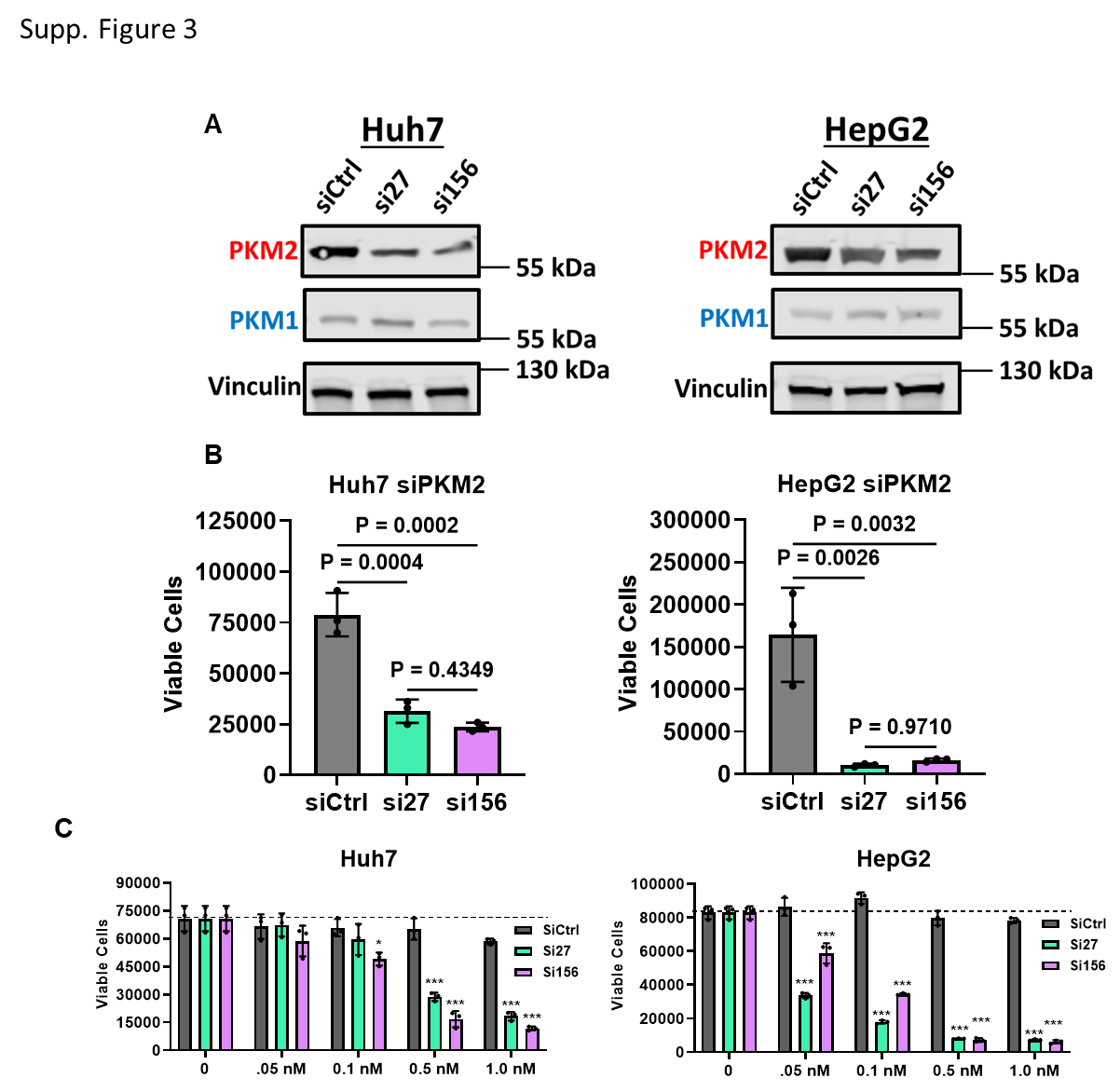
**Supplemental Figure 3. siRNA knockdown of PKM2 inhibits HCC cell proliferation*.*** (A) Western blotting analysis of PKM isoforms in Huh7 (left) and HepG2 (right) liver-cancer cell lines after transfecting 5 nM siRNA for 2 d. (B) Viable cells counted with flow cytometry. Cells were transfected with 5 nM siRNA and incubated for 5 d, with repeat transfection on day 2. (C) Viable cells counted and treated with various concentrations of siRNA as in (B). The bar graphs in (B) and (C) represent the average of three independent biological replicates ± SD; one-way ANOVA was performed with Tukey’s multiple comparison post-hoc test. For (C) statistical analysis was performed for each siRNA concentration displayed. * P < .05; ** P < .01; *** P < .001.

.


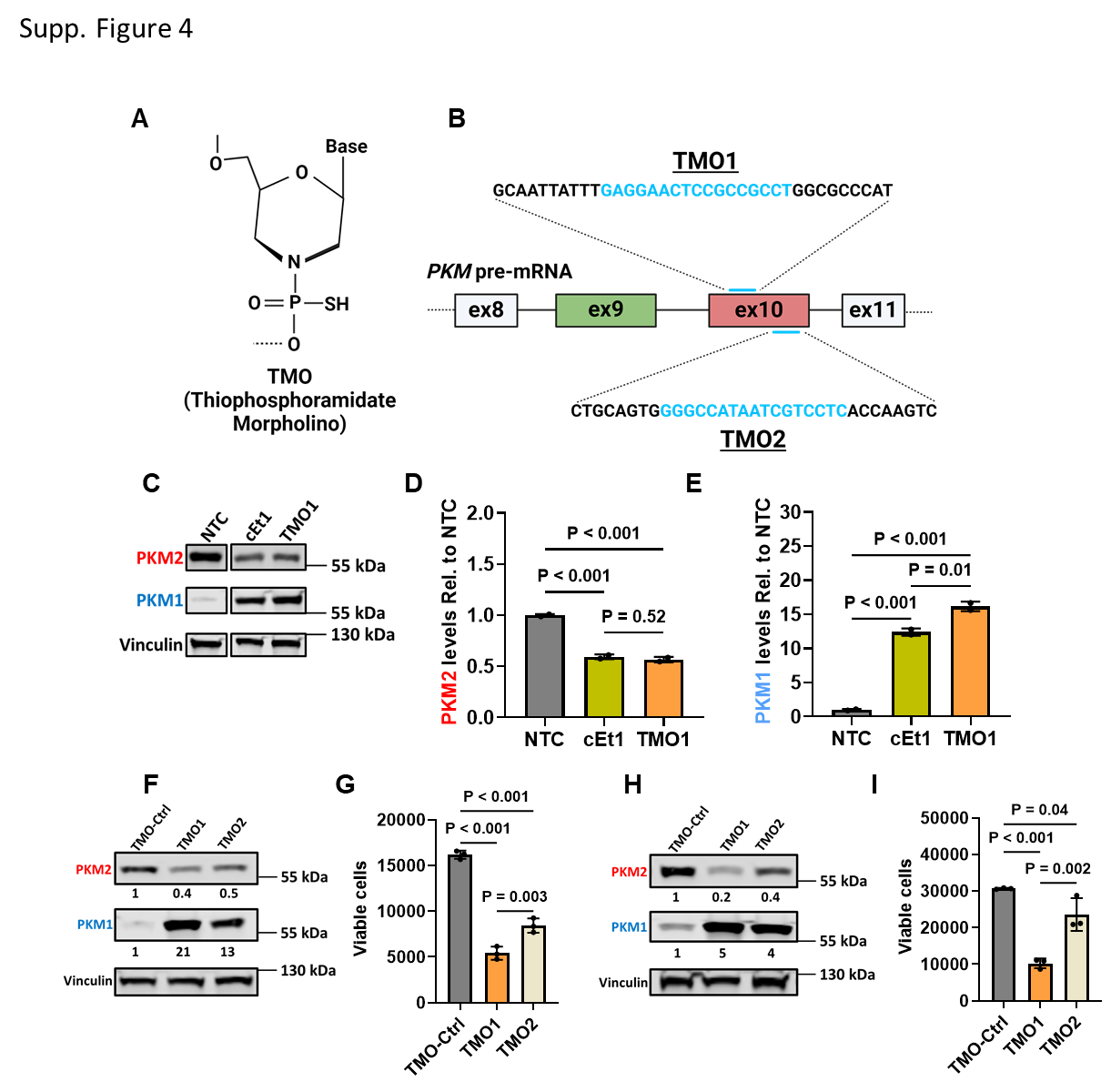
**Supplemental Figure 4. Comparison of *PKM* splice-switching ASOs in HCC cell lines.** (A) Chemical structure of the thiophosphoramidate morpholino (TMO) modification. (B) Diagram depicting the target binding region of TMO1 (above) and TMO2 (below) on *PKM* exon 10. (C) Representative western blotting analysis of the PKM isoform switch in HepG2 cells after transfection for 48 h with 60 nM of the indicated ASO. (D) and (E) Quantification of band intensities from (C); bands were normalized to vinculin and to the no-transfection control (NTC). (F) Representative western blotting analysis of the PKM isoform switch in Huh7 cells transfected with 60 nM TMO1 or TMO2 for 4 d. Quantification of band intensities is shown below; bands were normalized to vinculin and to the TMO-Ctrl. (G) Viable cells counted with flow cytometry after treatment as in (F). (H) and (I), Western blotting analysis and viable cell counts from SNU449 cells treated and analyzed as in (F) and (G). One-way ANOVA was performed with Tukey’s multiple comparison post-hoc test for data in (D), (E), (G), and (I). Data in (D) and (E) are from two independent biological replicates. The bar graphs in (G) and (I) represent the average of three independent biological replicates ± SD.

**
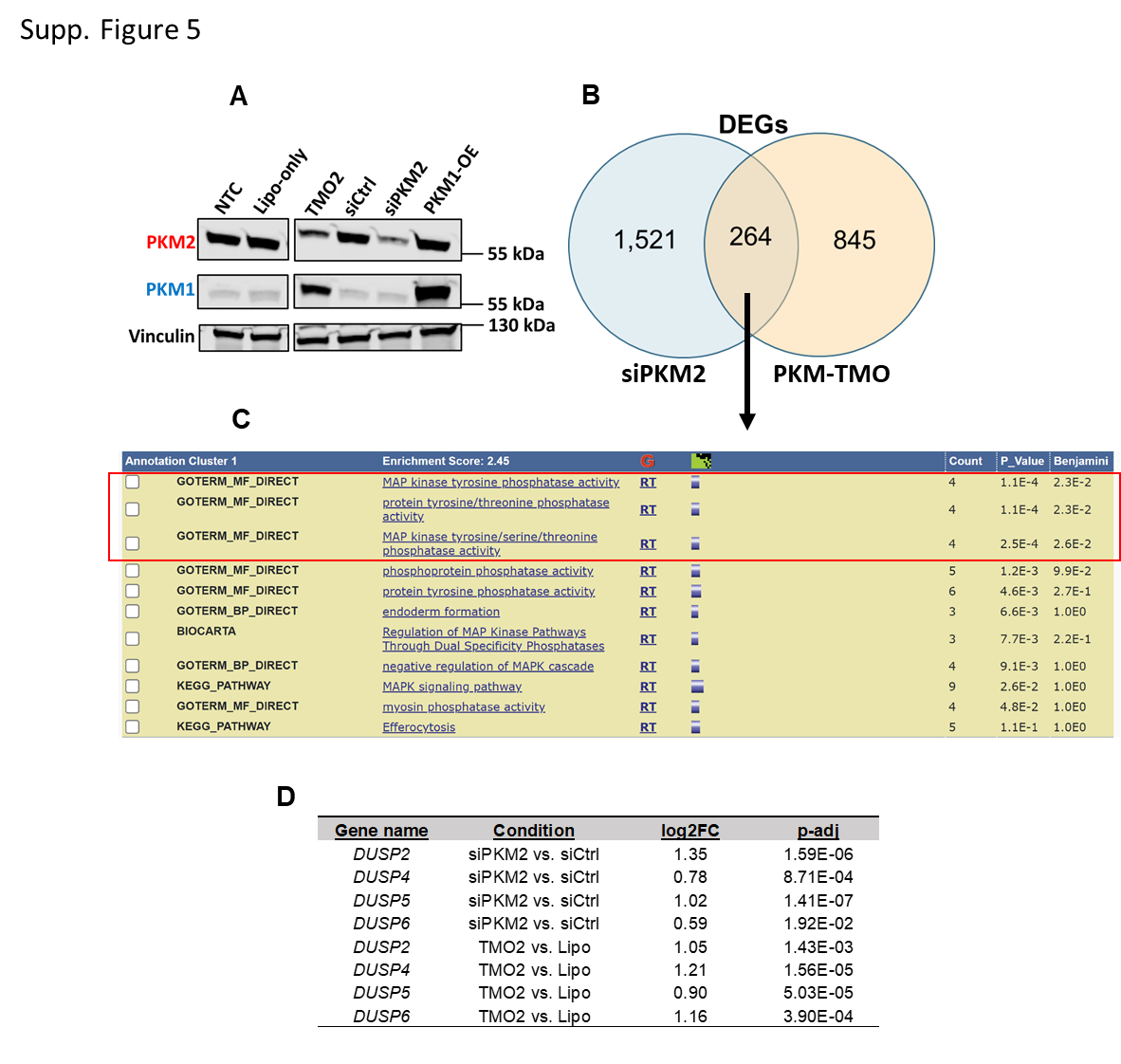
****Supplemental Figure 5. RNA-seq analysis of SNU449 cells treated with siPKM2 or PKM-TMO reveals increased expression of multiple DUSPs.** (A) Representative western blotting analysis of PKM isoforms in the samples used for RNA-seq analysis. SNU449-PKM1OE cells were treated for 48 h with either transfection of 60 nM PKM-TMO, 5 nM siRNA, or 0.25 µg/mL dox. (B) Venn diagram depicting the comparison of various treatment groups and their differentially expressed genes (DEGs), which were used in DAVID to identify annotated pathways. (C) Snapshot of results from DAVID after entering the list of shared DEGs shown in (B). (D) List of significantly upregulated DUSPs identified in the RNA-seq analysis, in cells treated with either siPKM2 or PKM-TMO2. Data in (B-D) represent the average of three independent biological replicates, and were analyzed as described in the Experimental Procedures for RNA-seq.

.

**
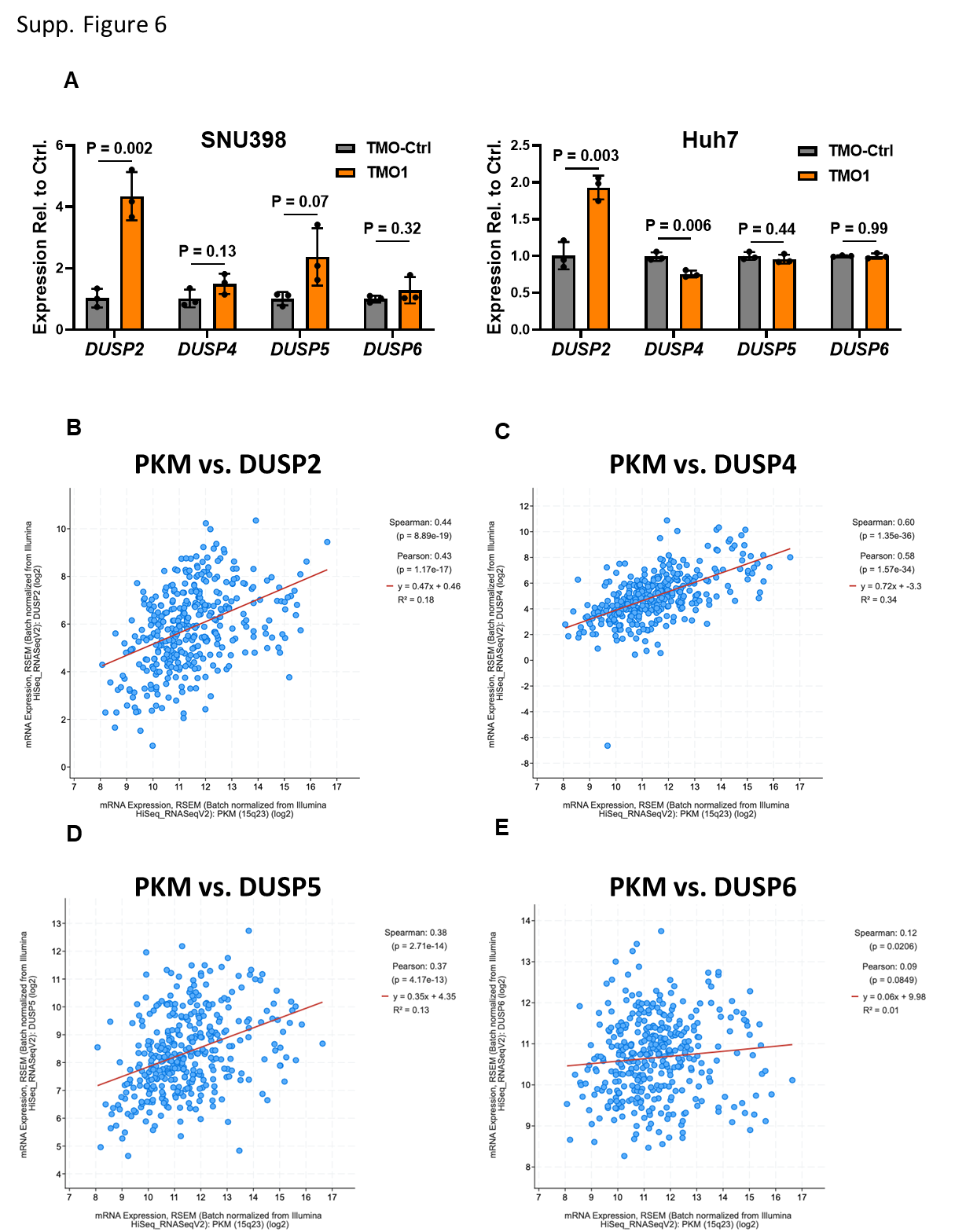
**

**Supplemental Figure 6. Expression of *PKM* and various *DUSP* genes in HCC tumors.** (A) RT-qPCR quantitation shows the extent of *DUSP* mRNA increase following transfection of either SNU398 (left) or Huh7 (right) cells with 60 nM TMO1 for 48 h. All tested transcripts were normalized to the *ACTB* mRNA level. Relative expression to cells treated with TMO-Ctrl is plotted. (B-E) Scatter plots of *PKM* versus the indicated *DUSP* expression level in HCC tumors, which were created using cBioPortal (version 6.0.8, TCGA-LIHC, PanCancer Atlas). The bar graph in (A) represents the average of three independent biological replicates ± SD. In (A), a two-sided t-test was performed for each time point shown. For data in (B-E), Spearman's rank correlation was used to determine the relationship between *PKM* and each *DUSP*.


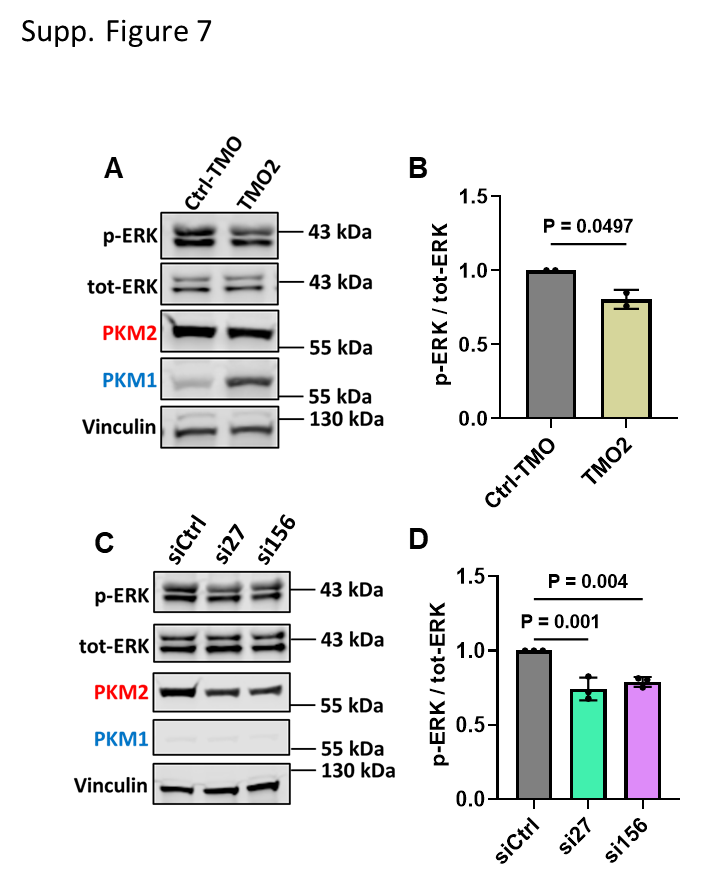


**Supplemental Figure 7. Downregulation of PKM2 via siPKM2 or PKM-TMO results in dephosphorylation of ERK1/2.** (A) Representative western blotting analysis of phosphorylated and unphosphorylated ERK1/2 (p-ERK and tot-ERK, respectively), and PKM isoforms, in SNU449 cells transfected with 60 nM of TMO2 for 48 h. (B) Quantification of the ratio of band intensities for p-ERK and tot-ERK from (A); bands were normalized to vinculin, then p-ERK was normalized to tot-ERK, and this ratio was then normalized to Ctrl-TMO. (C) Representative western blotting analysis of proteins as in (A) from SNU449 cells transfected with 5 nM si27 or si156 for 48 h. (D) p-ERK and tot-ERK bands were normalized as in (B) and the ratio was then normalized to siCtrl. The bar graphs in (B) and (D) represent the average of three independent biological replicates ± SD, and a two-sided *t*-test was performed for each graph.
